# Herpes DNAemia and TTV Viraemia in Intensive Care Unit Critically Ill Patients: A Single-Centre Prospective Longitudinal Study

**DOI:** 10.3389/fimmu.2021.698808

**Published:** 2021-11-02

**Authors:** François Mallet, Léa Diouf, Boris Meunier, Magali Perret, Frédéric Reynier, Philippe Leissner, Laurence Quemeneur, Andrew D. Griffiths, Virginie Moucadel, Alexandre Pachot, Fabienne Venet, Guillaume Monneret, Alain Lepape, Thomas Rimmelé, Lionel K. Tan, Karen Brengel-Pesce, Julien Textoris

**Affiliations:** ^1^EA 7426 “Pathophysiology of Injury-Induced Immunosuppression” (Université Claude Bernard Lyon 1—Hospices Civils de Lyon—bioMérieux), Joint Research Unit HCL-bioMérieux, Immunology Laboratory & Anesthesia and Critical Care Medicine Department, Hospices Civils de Lyon, Edouard Herriot Hospital, Lyon, France; ^2^IVIDATA, Levallois-Perret, France; ^3^Soladis Inc., Cambridge, MA, United States; ^4^BIOASTER Technology Research Institute, Lyon, France; ^5^Sanofi Pasteur, Sanofi, Marcy l’Etoile, France; ^6^Laboratoire de Biochimie (LBC), École Supérieure de Physique et de Chimie Industrielles de la ville de Paris (ESPCI) Paris, Paris Sciences & Lettres (PSL) Université, Centre National de la Recherche Scientifique (CNRS) UMR8231, Paris, France; ^7^GlaxoSmithKline, Brentford, United Kingdom

**Keywords:** herpesviruses, TTV, intensive care unit, sepsis, mortality, acquired infection, immune response, biomarkers

## Abstract

**Introduction:**

We analysed blood DNAemia of TTV and four herpesviruses (CMV, EBV, HHV6, and HSV-1) in the REAnimation Low Immune Status Marker (REALISM) cohort of critically ill patients who had presented with either sepsis, burns, severe trauma, or major surgery. The aim was to identify common features related to virus and injury-associated pathologies and specific features linking one or several viruses to a particular pathological context.

**Methods:**

Overall and individual viral DNAemia were measured over a month using quantitative PCR assays from the 377 patients in the REALISM cohort. These patients were characterised by clinical outcomes [severity scores, mortality, Intensive Care Unit (ICU)-acquired infection (IAI)] and 48 parameters defining their host response after injury (cell populations, immune functional assays, and biomarkers). Association between viraemic event and clinical outcomes or immune markers was assessed using χ^2^-test or exact Fisher’s test for qualitative variables and Wilcoxon test for continuous variables.

**Results:**

The cumulative incidence of viral DNAemia increased from below 4% at ICU admission to 35% for each herpesvirus during the first month. EBV, HSV1, HHV6, and CMV were detected in 18%, 12%, 10%, and 9% of patients, respectively. The incidence of high TTV viraemia (>10,000 copies/ml) increased from 11% to 15% during the same period. Herpesvirus viraemia was associated with severity at admission; CMV and HHV6 viraemia correlated with mortality during the first week and over the month. The presence of individual herpesvirus during the first month was significantly associated (p < 0.001) with the occurrence of IAI, whilst herpesvirus DNAemia coupled with high TTV viraemia during the very first week was associated with IAI. Herpesvirus viraemia was associated with a lasting exacerbated host immune response, with concurrent profound immune suppression and hyper inflammation, and delayed return to immune homeostasis. The percentage of patients presenting with herpesvirus DNAemia was significantly higher in sepsis than in all other groups. Primary infection in the hospital and high IL10 levels might favour EBV and CMV reactivation.

**Conclusion:**

In this cohort of ICU patients, phenotypic differences were observed between TTV and herpesviruses DNAemia. The higher prevalence of herpesvirus DNAemia in sepsis hints at further studies that may enable a better *in vivo* understanding of host determinants of herpesvirus viral reactivation. Furthermore, our data suggest that EBV and TTV may be useful as additional markers to predict clinical deterioration in ICU patients.

## Introduction

Viral reactivation in critically ill non-immunocompromised patients may have important clinical implications. Despite many well-conducted clinical trials, it remains unclear whether these viruses are at best innocent but informative bystanders or at worst pathogens requiring pre-emptive treatment (1–4). Current dogma is that the reactivation of latent viruses is one of the consequences of immunosuppression, although no cause and effect relationship has been demonstrated to date. Immunosuppression following an initial proinflammatory response to injury (sepsis, trauma, etc.) is frequent in non-immunocompromised Intensive Care Unit (ICU) patients (5). Several studies have demonstrated a significant association between immune alterations and an increased incidence of secondary infections [reviewed in (6)], and a recent study highlighted that the persistence of profound immune alterations in 20% of ICU patients at the end of the first week was independently associated with the risk of secondary infection (7).

Torque teno virus (TTV), the most abundant component of the human virome (8), was detected in adult (9, 10) and paediatric patients with sepsis (11). Moreover, herpesviruses (e.g., CMV, EBV, HHV6, HSV1) have also been shown to be reactivated in adult (9, 10, 12, 13) and in paediatric patients with sepsis (11), and in ostensibly immunocompetent critically ill patients (14, 15). EBV reactivation in sepsis was associated with an increased risk of mortality (10, 13) and was also observed in a diverse group of ICU patients (14). Recently, detection of EBV in patients with sepsis due to community-acquired pneumonia was associated with sepsis response signature (SRS) endotype, previously shown to be associated with increased mortality and features of immunosuppression (16). Similarly, an increase ICU mortality was associated with the detection of HSV-1 (17, 18) and CMV (19), whilst CMV detection in ICU patients was also associated with nosocomial infections (19). Furthermore, concomitant reactivation of several herpesviruses was associated with worse clinical outcomes than reactivation of a single virus alone (10, 20). Nevertheless, the correlation of herpesvirus reactivation with the changes in the immune system during an ICU stay remains a challenge.

In order to better understand the pathophysiology underlying viral reactivation in ICU patients, we choose to analyse blood DNAemia of CMV, EBV, HHV6, HSV-1 herpesviruses, and TTV anellovirus over 1 month of follow-up in the REAnimation Low Immune Status Marker (REALISM) study, which evaluated the evolution of the immune response of 377 patients admitted to the ICU due to sepsis, burns, trauma, and major surgery (21). The current ancillary study aimed to identify common features related to these viruses in the ICU population and characterise features linking one or several viruses to a particular pathological context. Association of virological markers with clinical outcomes and various immunological parameters were analysed in order to better define the causes and consequences of viral reactivation.

## Materials and Methods

Detailed study design including ethics and experimental protocols has been published previously (7, 10, 21). The key aspects are summarised below.

### Patient Cohort and Healthy Controls

We performed a prospective, observational cohort study of critically ill patients who presented with either sepsis, severe trauma, severe burns, or planned surgery to the Anesthesiology and Intensive Care Medicine Department of the Edouard Herriot Hospital (Hospices Civils de Lyon, France) during a 28-month inclusion period (December 2015–March 2018). The study protocol was approved by the institutional ethical review board (Comité de Protection des Personnes Sud-Est II) under number 2015-42-2. This clinical study was also registered at clinicaltrials.gov (NCT02638779). Written informed consent was obtained from every healthy volunteer and patient upon inclusion. If a patient was unable to consent directly, informed consent was obtained from the patient’s legally authorised representative and reconfirmed from the patient at the earliest opportunity. Inclusion criteria were as follows: patients aged >18 years, clinical diagnosis of sepsis as defined by 2016 SEPSIS-3 consensus guidelines (22), severe trauma with injury severity score (ISS) >15, severe burn with a total burn surface area over 30% or surgical patients undergoing major surgery such as oesophago-gastrectomy, bladder resection with Brickers’ reconstruction, cephalic pancreaticoduodenectomy, and abdominal aortic aneurysm surgery by laparotomy. Exclusion criteria were any of the following: presence of a pre-existing condition or treatment that could influence a patient’s immune status, e.g., corticosteroids or chemotherapy; pregnancy; institutionalised patients; or an inability to obtain informed consent (21). A cohort of 175 healthy volunteers aged 18–82 years (81 male and 94 female) were also prospectively recruited. To account for the possible influence of age and sex on immune parameters, the distribution of healthy volunteers was based on the age and sex demographic data for the French population in 2016.

### Sampling and Data Collection

In all patients, clinical samples and data were collected three times during the first week after admission: at day 1 or 2 (D1–2), D3 or 4 (D3–4), and D5, D6, or D7 (D5–7) and two times during the rest of the month, at D14 and D28. One sample was collected in healthy volunteers during the study visit, and clinical data were recorded. Data collection was detailed elsewhere (7). Patient demographics, comorbidities, diagnosis, severity, and clinical outcome were manually curated in a prospective fashion. Longitudinal follow-up was performed for a 30-day period. Peripheral whole blood collected from each patient and healthy volunteer at each time point was processed within 3 h after blood sampling. Blood was collected in one ethylenediaminetetraacetic acid (EDTA) tube for plasma viral DNAemia determination, flow cytometry immune phenotyping, and plasma cytokine level measurements; two heparin tubes for functional tests (proliferation, cytokine production experiments); and one PAXgene blood RNA tube (PreAnalytix, Hilden, Germany) for whole blood biomarkers mRNA concentration measurements by real-time PCRs using Eva-Green or TaqMan probe methodologies. PAXgene samples were stabilised for at least 2 h at room temperature after collection and frozen at −80°C following manufacturer’s recommendations.

### Definitions of Outcomes

The main outcomes were 28-day (D28) secondary infection and D28 mortality. Additional outcomes included ICU length of stay (LOS) and total hospital LOS, daily use of an invasive medical device (tracheal intubation, indwelling urinary catheter, and central venous line), and the incidence of secondary infections. Information collected about infections was reviewed by an independent adjudication committee composed of three clinicians not involved in study patients’ recruitment or care. Confirmation of the occurrence of secondary infection by this committee was based on guidelines of the European Society of Clinical Microbiology and Infectious Diseases and the Infectious Diseases Society of America. Only the first episode of secondary infection was considered in the analyses.

### Viral DNAemia Determination

The semi-automated process of viral DNAemia determination from four herpesviruses and TTV, coupling sample treatment, and quantitative real-time PCR standardised procedures were previously described in detail (10). Briefly, extraction of viral DNA from plasma was undertaken using Maxwell^®^ HT Viral TNA chemistry (Promega) consisting of paramagnetic silica particles and the liquid handling robot Freedom EVO^®^ (TECAN). PCR controls were spiked into each plasma sample prior to extraction. Limit of detection (LOD) was previously determined in the semi-automated process as the lowest quantity that can be distinguished from the absence of detection (a blank value) using viral standards (10). LOD expressed in copies/ml of plasma was 100 for CMV, 33 for EBV, 166 for HHV6, 33 for HSV1, and 167 for TTV. DNA viruses real-time PCR reactions were performed on the StepOnePlus™ Real-Time PCR System (Thermo Fisher Scientific) using TaqMan probe-based R-GENE^®^ assay kits for CMV, EBV, HSV1, HHV6, and TTV (bioMérieux SA). All nucleic acid samples, randomly batched in plates, were simultaneously amplified with quantification standards and sensitivity and negative controls, according to the manufacturer’s instructions. Finally, using standard curves, sample CT values were converted into copies/µl of viral DNA in the PCR reaction tube, then in copies/ml of viral DNA in the plasma.

### Serology

Enzyme-linked immunosorbent assay (ELISA) was used to test for the presence of IgG against the CMV and HSV1 viral capsid antigen (VCA; Abcam, Cambridge, UK) according to the manufacturer’s instructions. Plasma samples (10 µl) were diluted to the recommended 1:100 concentration and run in duplicate. Absorbance was measured at 450 nm using the CLARIOstar plate reader (BMG Labtech, Ortenberg, Germany). Serological evaluation was not undertaken for EBV, HHV6, and TTV.

### Cells, IFA, Cytokines, and Transcriptomic Markers

The complete list of the immune profiling panel reflecting complementary aspects of both innate and adaptive arms has been detailed elsewhere (21). Briefly, it included cellular markers [neutrophils, monocytes, natural killer (NK) cells, and lymphocytes], functional markers [cytokine production, immune functional assays, and human leukocyte antigen DR isotype (HLA-DR) density], and transcriptomic markers (23–26) measured from the blood. Only the following markers that were found to be associated with at least one viraemic episode are depicted: (i) cell populations detected by flow cytometry (% of immature (CD16^low^CD10^low^) and mature (CD16^high^CD10^high^) neutrophils, NK cell count (cells/µl), % of regulatory T cells (CD4^+^CD25^high^CD127^Low^), T lymphocyte count (cells/µl), T CD4+ lymphocyte count (cells/µl), T CD8+ lymphocyte count (cells/µl), and B lymphocyte count (cells/µl); (ii) functional markers and immune reactivity [tumor necrosis factor alpha (TNFα) release after lipopolysaccharide (LPS) stimulation, interferon gamma (IFNγ) release after Staphylococcal enterotoxin B (SEB) stimulation, plasma interleukin (IL)-6 and IL-10 concentrations, HLA-DR on monocytes and on B lymphocytes]; and (iii) transcriptomic markers (*BTLA, C3AR1, CD40L, HERVE4.1, IFIH1, IL15, LTR101, LTR33 OAS3, LTR40A, MER4B APOL3, MER50C, MLT1I, MLT2B5, MSTC, STAT4, IL7R, CD127s, CD30, CD74, CTLA4, CX3XR1, ICOS, IFNγ, IL10, IL18, IL18R1, IL18RAP, IL1R2, IL1RN, OAS3, TNFSF4, PD-1, S100A9*, and *HP*).

### Statistical Analysis

Within the first week after ICU admission, for each virus and for each patient, early viraemia was defined as positive if at least one of the patient’s samples (day 1/2, day 3/4, or day 5/7) was positive. Over a month after admission, viraemia was defined as positive if at least one of the patient’s samples was positive on day 1/2, day 3/4, day 5/7, day 14, or day 28. The positivity was set by the level of a virus copies/ml of plasma above a pre-defined threshold (LOD, see above). Data are presented as numbers and percentages (qualitative variables) and medians and 25th/75th percentiles (quantitative variables).

Association between viraemic event and clinical outcomes [survival or ICU acquired bacterial infection as previously described (10, 23)] was assessed using χ^2^-test or exact Fisher’s test. Association between binary DNAemia/viraemic event and quantitative immune markers (cells, cytokines, and mRNA) was assessed using Wilcoxon rank-sum test. Analyses were conducted with R version 3.4.4, and statistical significance was defined by an alpha-risk of falsely rejecting the null hypothesis of 5% (p < 0.05 indicates a significant association).

## Results

### Cohort Description

We screened the REALISM cohort of 377 critically ill patients: 107 sepsis, 137 severe trauma, 24 severe burns, and 109 major surgery. At ICU admission, serological testing (IgG) against CMV and HSV-1 antigens was used to determine whether viraemia was a primary viral infection or secondary to reactivation. The CMV-IgG positive rate ranged from 45% in severe trauma to 61% in sepsis and major surgery patients. The HSV1-IgG positive rate ranged from 56% in severe trauma to 78% in sepsis patients. Of the 206 patients with positive CMV-IgG, 34 (18%) also had CMV DNAemia indicating viral reactivation, whilst no CMV DNAemia was detected in 151 patients with negative CMV-IgG. Of the 251 patients with HSV1-IgG, 39 (16%) also had an HSV1 DNAemia, suggesting viral reactivation; however, 4/107 with negative HSV1-IgG had HSV1 DNAemia, representing 3.7% primary HSV1 infection rate. Although serology was not performed for the other viruses, these results suggested that herpesvirus-positive DNAemia largely resulted from viral reactivation.

Quantitative (viral load) rather than qualitative (DNAemia) data may enable better discrimination between (1) non-significant viral load, (2) viral “reactivation” as a putative marker of immunosuppression, and (3) high viral loads supporting a true viral infection requiring treatment (27).

Herpesvirus viral titres >10,000 copies/ml (considered to be high viral load supporting a true viral infection) were present in only 7% of HSV1, 6% of CMV, 3% of HHV6, and none of EBV patients that presented with positive herpesvirus DNAemia (**Supplementary Figure S1**). Herpesviruses DNAemia were mostly discrete, single events (from 56% to 85% of unique viral events over the period) rather than persistent (from 2% to 24% of three or more viral events over the period). Thus, Herpesviridae viraemia was subsequently only analysed as the presence of DNAemia and viral load was not analysed further. As pleiotropic herpesviruses may develop lifelong latency with reactivation during periods of immunosuppression (28–30), we first considered Herpesviridae-related viraemia as a whole, i.e., herpesviruses were grouped as a surrogate of immune failure. Of the 377 patients, 132 (35%) had at least one Herpesviridae-related viraemia during the first month (**Table 1**) with 96/377 (25%) during the first week (**Supplementary Table S1**). Patients with Herpesviridae-related viraemia were more severely unwell (**Table 1**), with higher severity scores, higher plasma lactate level, increased need for mechanical ventilation and supportive treatments (hydrocortisone, dopamine, or noradrenaline) and with worse outcomes (based on length of stay, mortality, and ICU-acquired infection). Viral reactivation was more associated with sepsis compared to other pathologies (p < 0.001) and individuals with hospital-acquired infection compared to community-acquired infection (p = 0.047).

**Table 1:** ICU patient characteristics at admission and outcomes according to herpesvirus viral DNAemia during the first month (D1–D28) in ICU (excluding TTV).

	Viremia not present (n = 245)	At least one herpes viremia (n = 132)	Whole cohort (n = 377)	p-value
**Demographics**				
Age years	54.5 [44–69]	62.8 [53–75]	57.4 [47–71]	<0.001***
Gender (female)	81 (33%)	48 (36%)	129 (34%)	0.519
**Pathologies**				<0.001***
Sepsis	47 (19%)	60 (45%)	107 (28%)	
Trauma	111 (45%)	26 (20%)	137 (36%)	
Surgery	78 (32%)	31 (23%)	109 (29%)	
Burn	9 (4%)	15 (11%)	24 (6%)	
**Admission**				
SAPSII day 1	28 [18–37]	39.8 [27.8–51]	32.1 [20–44]	<0.001***
SOFA score day 1	4.2 [1–8]	6.7 [3–10]	5.1 [1–8]	<0.001***
Charlson score day 1	1.3 [0–2]	1.9 [0–3]	1.5 [0–2]	0.001**
**Sepsis primary infection type**				0.047*
Community acquired	38 (16%)	38 (29%)	76 (20%)	
Hospital acquired	9 (4%)	22 (17%)	31 (8%)	
**Sepsis primary infection site**				0.574
Abdominal	18 (7%)	24 (18%)	42 (11%)	
Pulmonary	10 (4%)	17 (13%)	27 (7%)	
Others	19 (8%)	19 (14%)	38 (10%)	
**Treatment**				
Hydrocortisone	17 (7%)	24 (18%)	41 (11%)	0.001**
Vasopressors day 1	107 (44%)	90 (68%)	197 (52%)	<0.001***
**Chemistry and haematology at admission**				
Monocytes (G/L)	1.1 [0.7–1.3]	1.1 [0.6–1.3]	1.1 [0.7–1.3]	0.176
Lymphocytes (G/L)	1.4 [0.8–1.8]	1.4 [0.8–1.8]	1.4 [0.8–1.8]	0.831
Neutrophils (G/L)	12.2 [8.4–14.6]	13.2 [7.4–16]	12.6 [8–15.1]	0.909
Lactate concentration (mM)	2.5 [1.7–3.2]	3.2 [1.9–3.8]	2.8 [1.7–3.4]	0.048*
**Outcomes**				
Haemodialysis duration (days)	11.9 [1.2–6.5]	13.7 [2–20.5]	13.2 [1.8–14.5]	0.27
Mechanical Ventilation (days)	6.1 [1–4]	11.5 [1–13.8]	8.8 [1–9]	0.001**
ICU length of stay (days)	8.8 [3–9]	16.3 [5–18]	11.6 [4–12.2]	<0.001***
Hospital length of stay (days)	18.2 [8–23]	28.4 [13–43]	21.6 [9–27]	<0.001***
ICU Mortality D28	7 (3%)	14 (11%)	21 (6%)	0.002**
At least one IAI D28	46 (19%)	51 (39%)	97 (26%)	<0.001***

Categorical variables are expressed as n (%) and continuous variables as median [Q1–Q3]. Comparisons between detected or not detected herpesviruses (CMV, EBV, HHV6, and HSV1), were performed with a chi-squared test for qualitative variables and Wilcoxon test for quantitative variables, as appropriate. p-values with stars indications represent significance at p < 0.05.

ICU, Intensive Care Unit; IAI, ICU-acquired infection; SOFA, sequential organ failure assessment; SAPS, Simplified Acute Physiology Score.

In contrast to herpesvirus-positive individuals, among TTV DNA-positive individuals, 32% had TTV viral titres >10,000 copies/ml (**Supplementary Figure S1**), as compared to 10% of healthy volunteers. Additionally, TTV DNAemia was mostly frequently persistent (70%) rather than discrete single DNAemia events (15%) unlike the herpesviruses (**Supplementary Figure S1**); however, <6% of TTV-positive patients presented with significant fivefold variations in viral load. Thus, TTV viraemia was subsequently only analysed as the presence of DNAemia. Of the 377 patients, 228 (60%) had TTV-related viraemia during the first month with 217/377 (58%) during the first week (**Supplementary Table S2**). Patients with higher TTV viraemia were more severely unwell, with higher severity scores and increased need for supportive treatments (**Table 2**). A male sex bias has been described for TTV (31, 32), and this was also observed in the current study. This bias was significantly more marked in patients with TTV titres above 10,000 copies/ml (henceforth referred to as TTVh). We selected this threshold to further explore TTV viraemia in ICU patients.

**Table 2:** ICU patient characteristics at admission and outcomes according to TTV plasma DNAemia above 167 copies/ml (LOD), 7,000, 10,000, and 40,000 copies/ml, during the first month (D1–D28) in ICU.

	TTV >167 cp/ml	p-value	TTV >7,000 cp/ml	p-value	TTV >10,000 cp/ml	p-value	TTV >40,000 cp/ml	p-value
	(n = 228)		(n= 75)		(n = 57)		(n = 29)	
**Demographics**								
Age years	58.8 (47−73]	0.026*	59.8 (52−71.5]	0.114	60.7 (53−73]	0.077	62.4 [56−75]	0.086
Gender (female)	75 (33%)	0.503	20 (27%)	0.124	13 (23%)	0.049*	5 (17%)	0.045*
**Pathologies**		0.421		0.408		0.334		0.392
Sepsis	64 (28%)		22 (29%)		17 (30%)		8 (28%)	
Trauma	83 (36%)		22 (29%)		15 (26%)		7 (24%)	
Surgery	70 (31%)		24 (32%)		20 (35%)		12 (41%)	
Burn	11 (5%)		7 (9%)		5 (9%)		2 (7%)	
**Admission**								
SAPSII day 1	32.1 [20−43.2]	0.871	35.9 [23−46]	0.037*	36.2 [25−46]	0.045*	35.4 [25−45]	0.266
SOFA score day 1	5 [1−8]	0.643	6 [2−9]	0.024*	6.2 [3−9]	0.021*	5.8 [3−8]	0.222
Charlson score day 1	1.6 [0−3]	0.105	2.1 [0−3]	0.021*	2.3 [0−4]	0.012*	2.8 [0−5]	0.002**
**Treatment**								
Hydrocortisone	27 (12%)	0.456	11 (15%)	0.239	10 (18%)	0.079	7 (24%)	0.017*
Vasopressors day 1	118 (52%)	0.81	47 (63%)	0.044*	37 (65%)	0.038*	21 (72%)	0.024*
**Chemistry and haematology at admission**								
Lymphocytes (G/L)	1.4 [0.8–1.8]	0.918	1.4 [0.9–1.8]	0.8	1.3 [0.8–1.7]	0.289	1.3 [0.9–1.7]	0.691
Lactate concentration (mM)	2.7 [1.7–3.3]	0.244	2.5 [1.6–3.3]	0.503	2.4 [1.6–3]	0.4	2.4 [1.8–3]	0.516
**Outcomes**							11 (38%)	
ICU length of stay (days)	11.5 [4–12]	0.611	14.1 [4–16]	0.13	11.8 [4–12]	0.825	13 [4–15]	0.648
Mortality D28	9 (4%)	0.089	4 (5%)	0.92	3 (5%)	0.913	0 (0%)	NA
At least one IAI D28	60 (26%)	0.747	23 (31%)	0.274	17 (30%)	0.443	11 (38%)	0.118

Categorical variables are expressed as n(%) and continuous variables as median [Q1–Q3]. Comparisons between detected (shown) or not detected (not shown) TTV were performed with a chi-squared test for qualitative variables and Wilcoxon test for quantitative variables, as appropriate. p-values with stars indications represent significance at p < 0.05.

NA, Not Applicable; SOFA, sequential organ failure assessment; SAPS, Simplified Acute Physiology Score.

### Viraemia in ICU Patients During the First Week and the First Month in ICU

**Figure 1:**
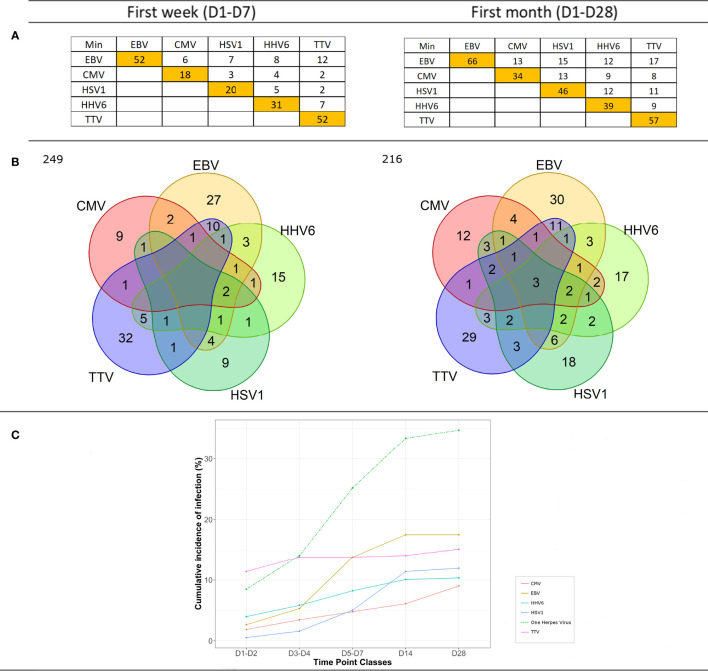
DNAemia during the first week and first month following admission. **(A)** Number of patients presenting single- or multiple-positive herpes and/or TTVh (>10,000 copies/ml) viral DNAemia during the first 7 days or the first 28 days following admission in the ICU. Occurrence of each type of herpesvirus (HV) or TTVh is depicted in the orange boxes. Co-occurrence of HV with other HV or TTVh is reported in white boxes. **(B)** Venn diagram illustrating single *versus* multiple viral reactivations; 121 then 94 patients presented with no viral event during the first week and the month, respectively. **(C)** Cumulative incidence of individual or collective HV and TTVh DNAemia at days 1–2, 3–4, 5–7, 14, and 28 following admission in the ICU.

The incidence of viral DNAemia at ICU admission was below 4% for each herpesvirus and 11% for TTVh. During the first week, among the 96 (25%) patients who had at least one Herpesviridae-related viraemia, 77 (20%) presented with one, 15 (4%) presented with two, and 4 (1%) presented with three or more different herpesviruses. In contrast, only one EBV and four HHV6 viraemias were detectable in 5/175 (3%) healthy volunteers. The most frequently detected herpesvirus in ICU patients was EBV (n = 52, 14%), followed by HHV6 (n = 31, 8%), HSV1 (n = 20, 5%), and CMV (n = 18, 5%) (**Figure 1A**). EBV and HHV6 were the most frequently observed herpesvirus co-infection (n = 8, 2%). There were also two occurrences of co-infection with all four herpesviruses (EBV, HHV6, HSV1, and CMV) simultaneously (**Figure 1B**). During the first week, TTVh was detected in 52 (14%) ICU patients. TTVh alone was detected in 32 (8%) patients, whilst TTVh was co-detected in patients with one (n = 17, 5%) or two (n = 3, 1%) herpesviruses. The most frequent herpesvirus co-detected with TTVh was EBV (n = 12, 3%) followed by HHV6 (n = 6, 2%), HSV1 (n = 2, <1%), and CMV (n = 2, <1%). Overall, the cumulative occurrence of herpesvirus DNAemia (**Figure 1C**) increased to 8%–25% (depending on the herpesvirus) during the first week, whilst TTVh DNAemia increased to 14%. Additionally, the occurrence of a single virus (n = 92), either herpesvirus or TTVh, was higher than the occurrence of at least two viruses (n = 36).

During the first month, among the 132 (35%) patients who had at least one Herpesviridae-related viraemia, 95 (25%) presented with one, 26 (7%) with two, and 11 (3%) with three or more different herpesviruses reactivation. The most frequently detected virus remained EBV (n = 66, 18%), followed by HSV1 (n = 46, 12%), HHV6 (n = 39, 10%), and CMV (n = 34, 9%) (**Figure 1A**). EBV (in combination with HSV1 or CMV or HHV6) (n = 15, 4%) and HSV1 (in combination with EBV or CMV or HHV6) (n = 15, 4%) were the most frequently observed herpesvirus co-infections. There were also five occurrences of co-infection with all four herpesviruses simultaneously (**Figure 1B**). TTVh was detected in 57 (15%) ICU patients. Twenty-nine (8%) patients expressed TTVh only, whilst TTVh was co-detected in patients with one (n = 18, 5%) and with two or more (n = 8, 2%) herpesviruses. All herpesviruses were co-detected with TTV, the most frequent being EBV (n = 17, 5%), then HSV1 (n = 11, 3%), HHV6 (n = 9, 2%), and CMV (n = 7, 2%). Overall, the cumulative occurrence of herpesvirus DNAemia (**Figure 1C**) increased to 8%–35% during the first month, whilst TTVh DNAemia increased to 15%. Additionally, the occurrence of a single virus (n = 106) remained higher than the increased occurrence of co-infection (n = 56).

### Association of Viraemia With Clinical Outcomes

Herpesvirus viraemia appeared to correlate with mortality during the first week and month of admission and also with the occurrence of ICU-associated infection (only over the month). Specifically, the presence of CMV or HHV6 from the first week in ICU appeared to correlate with mortality at D28 (**Table 3**). The overall mortality rate was low (4%); however, there was a 2.4 times increase in mortality between the presence of a single virus and the presence of at least two herpesviruses. The simultaneous presence of high TTV viral load and at least one herpesvirus appeared to be associated with a lower mortality, although this may be confounded by a lack of effect of TTV on mortality (**Table 3**). Detection of each individual herpesvirus, but not TTV, during the month was significantly associated with ICU-associated infection (IAI). Indeed, the presence of TTV alone (without any herpesvirus) appeared associated with a reduced occurrence of IAI (**Table 3**). In addition, during the first month, a stable TTV viral load appeared to be associated with a reduced occurrence of IAI (data not shown).

**Table 3:** Association between binary clinical outcomes and viremia presence.

Outcome (Otc)	Virus	Viremia first week^a^			Viremia month^a^		
		% viraemic patients		P value	% viraemic patients		P value
		Otc+^b^	Otc−^b^		Otc+^b^	Otc−^b^	
Mortality at D28^c^	One HpV	46.2	20.6	0.006**	53.9	27.0	0.046*
	At least one HpV	56.3	24.1	0.007**	62.5	33.8	0.029*
	At least two HpV	30.0	5.6	0.006**	33.3	12.5	0.046*
	CMV	18.8	4.2	0.035*	18.8	8.6	0.167
	EBV	18.8	13.6	0.472	18.8	17.5	1
	HHV6	31.3	7.2	0.006**	31.3	9.4	0.017*
	HSV1	12.5	5.0	0.206	18.8	11.9	0.427
	TTVh^e^	12.5	13.9	1	12.5	15.2	1
	TTVh only^f^	6.3	8.6	1	6.3	7.8	1
	TTVh^e^ and HpV^g^	6.3	5.3	0.590	6.3	7.5	1
IAI month^d^	One HpV	25.0	19.0	0.384	40.3	22.8	<0.001***
	At least one HpV	28.9	22.3	0.213	52.6	26.9	<0.001***
	At least two HpV	6.8	5.1	0.384	30.3	6.8	<0.001***
	CMV	3.1	4.5	0.768	18.6	4.9	<0.001***
	EBV	15.5	12.9	0.603	26.8	14.0	0.007**
	HHV6	11.3	5.7	0.105	16.5	6.8	0.008**
	HSV1	5.2	4.9	1	20.6	8.7	0.003**
	TTVh^e^	16.5	12.9	0.392	17.5	14.4	0.509
	TTVh only^f^	6.2	9.5	0.400	4.1	9.1	0.180
	TTVh^e^ and HpV^g^	10.3	3.4	0.015*	13.4	5.3	0.013*

Clinical outcomes are mortality at D28 following ICU admission and HAI occurrence occurring during the month of hospitalization.

a Viraemia detected D1–D7 (first week) and D1–D28 (month).

b Percent of patients with a detected viraemia according to the presence (Otc+ = death at 28 days or at least one IAI during the month) or absence (Otc− = no death at 28 days or absence of HAI during the month) of the outcome.

c Only 4% of death events during the month.

d 26% IAI (ICU acquired infection) events during the month.

e TTV DNAemia above 10,000 copies/ml.

f Exclusively TTV virus.

g At least one herpesvirus.

p values with stars indications represent significance at p < 0.05.

Concerning co-reactivation during the first month, patients with multiple viraemic events (two or more herpesvirus or one TTV and at least one herpesvirus) had a higher frequency of IAI (44%) compared to those with a viraemia from a single virus (22%) or patients with no viraemia (19%) (χ^2^-test, p <0.001). Meanwhile, patients with multiple viraemic events had a similar frequency of IAI episodes (11%) compared to those with either a viraemia from a single virus (9%) or no viraemia (9%) during the first week (χ^2^-test, p = 0.8). In contrast to herpes DNAemia alone, the co-detection of high titre TTV with herpesvirus during the first week of admission seemed indicative of the occurrence of ICU-associated infection (**Table 3**).

### Association of Viraemia With Immunological and Molecular Markers

**Figure 2:**
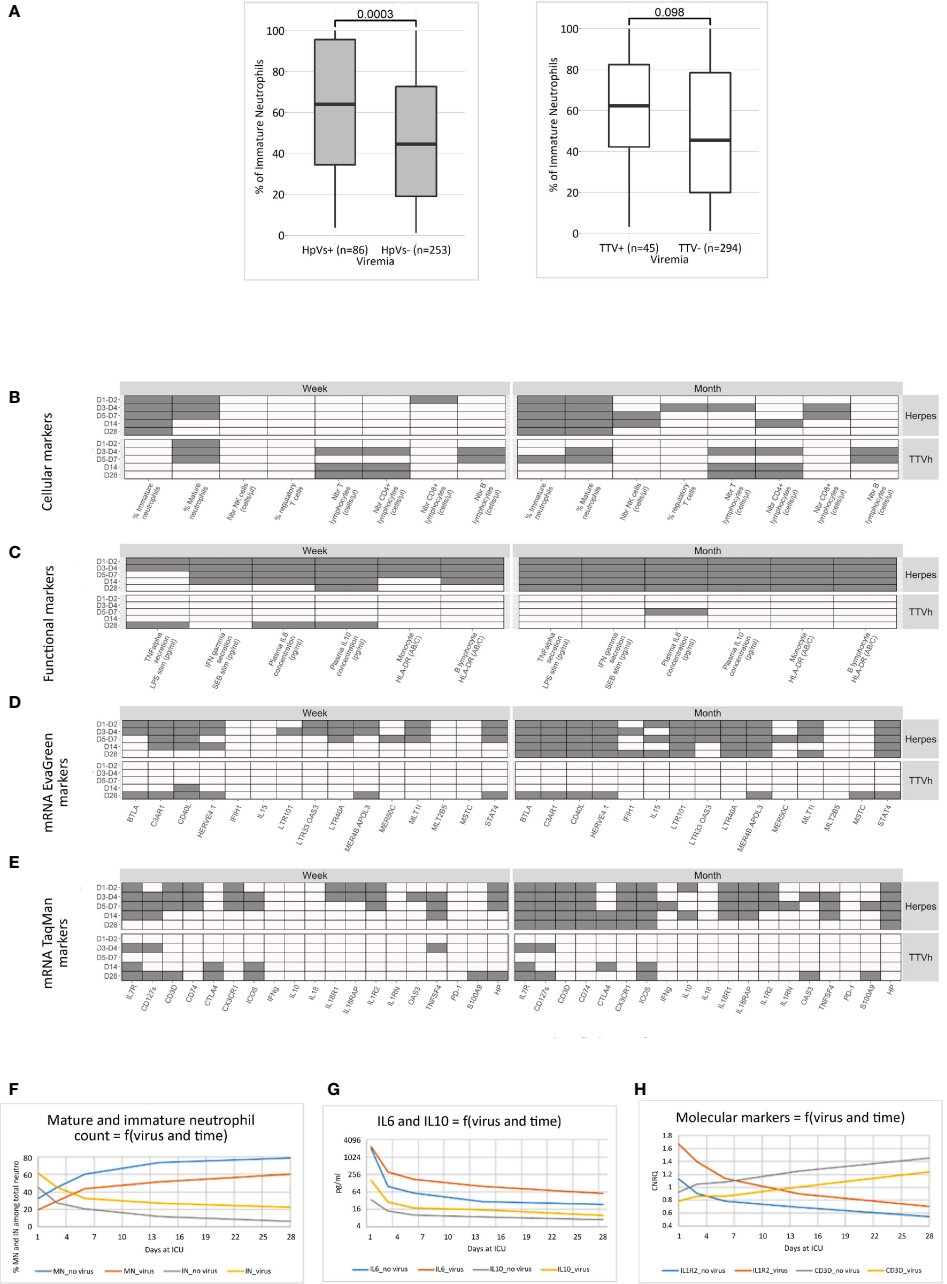
Association of viraemia with immunological and molecular markers. Wilcoxon tests among global pathological conditions were performed to compare markers distribution at different time points (D1–D2, D3–D4, D5–D7, D14, and D28) and viraemia (all Herpes or TTVh) detection status during the first week (D1–D7) and the first month (D1–D28). **(A)** Illustration of a significant (left) and a non-significant (right) association between viraemia during the first week (herpesviruses, left; TTVh, right) and immature neutrophil percentages at D1–D2. Significant associations are represented respectively by a grey box and non-significant associations by a white box in the following four charts **(B–E)**. Significant associations between viraemia and **(B)** cellular marker (flow cytometry), **(C)** functional markers (immune functional assay, plasma cytokine concentrations, cell-surface HLA-DR), **(D)** mRNA transcripts detected by RT-PCR_EVaGreen, and **(E)** mRNA markers detected by TaqMan RT-PCR are indicated as grey boxes. Comparative kinetics of six identified markers (mean expression in the whole cohort) was depicted as a function of time and the presence of herpes viremia in the D1-D28 period for **(F)** mature (CD10high_CD16high) and immature (CD10low_CD16low) neutrophils, **(G)** serum Il6 and IL10, and **(H)** IL1R2 an CD3D molecular markers.

To understand whether there was a correlation between the presence of these viruses and the immunological status of these patients, we undertook further immunological assessments, including evaluation of cellular populations, immune functional assays, cytokine production, HLA-DR expression, and molecular markers (**Figure 2A**). In order to identify the most relevant associations, we required at least three out of five associations between the marker variation across the five different time points (D1/2, D3/4, D5/7, D14, and D28) and the detection of viraemia to be significant; this was done both over the week and over the month. We identified mature and immature neutrophils (**Figure 2B**), HLA-DR per B lymphocyte and monocyte, IFN γ post-SEB stimulation, serum IL-6 and IL10 (**Figure 2C**), and a set of 14 out of 34 molecular markers (**Figures 2D, E****)** as being most informative.

Cellular and functional markers with a consistently higher count or expression in patients than in healthy volunteers were found to have an even higher level in patients presenting with herpesvirus plasma viraemia and vice versa. Over the period, individuals with herpesvirus reactivation had between 1.3 and 1.6 times fewer mature neutrophils and 1.4–3.5 times more immature neutrophils than those who had no detectable viraemia (**Figure 2F**). Individuals with viral reactivation had (i) between 1.1 and 1.3 times less HLA-DR per B lymphocyte and up to 1.5 times less HLA-DR per monocyte than those who had no detectable viraemia; (ii) between 1.1 and. 2 times less IFNγ release post SEB stimulation than those who had no detectable viraemia; and (iii) between 1.2 and 3.4 times more IL-6 and 1.4–4.7 times more IL10 in serum than those who had no detectable viraemia (**Figure 2G**).

Genes with a consistently higher expression in patients than in healthy volunteers, namely, *C3AR1*, *IL1R2* (**Figure 2H**), and *HP*, had higher expression in patients presenting with viraemia. In contrast, genes with a consistently lower expression in patients than in healthy volunteers, namely, *CD40L* (*CD154*), *STAT4*, *IL7R* (*CD127*), *CD127s*, *CD3D* (**Figure 2H**), *CD74*, and *CX3CR1*, had an even lower expression in patients with viraemia. In addition, the expression of some retrotransposons previously associated with immunocompromised status and disease severity (24, 33) was found to be associated with viraemia, namely, *HERVE 4.1* (170369402HE41env; lower expression with worsening of disease, lower expression with viraemia), *LTR40A* (050286701-HERV0513; higher expression with worsening of disease, higher expression with viraemia), and *MLT1I* (021456001-MALR1017; higher with worsening, higher expression with viraemia).

We used the same procedure to identify whether a single virus may account for the overall herpesvirus association with immunological markers (**Supplementary Figure S2**). Individuals who went on to develop EBV or HHV6 reactivation have more immature neutrophils than those who have no detectable viraemia. Only individuals who develop EBV reactivation had (i) less HLA-DR per B lymphocyte and per monocyte; (ii) less IFNγ-release post SEB stimulation and less TNFα-release post LPS stimulation; and (iii) more serum IL6 and IL10, than those who have no detectable viraemia. In addition, some immunological phenomenon were observed with specific viruses only; for example, individuals who develop CMV reactivation had less CD4+ lymphocyte than those who have no detectable viraemia, and those with HSV1 had lower T cell and notably CD8+ lymphocyte counts. Some genes previously unidentified in the overall analysis seemed associated with specific viraemia, such as *BTLA*, *ICOS*, and *MER4B_APOL3* upregulated in presence of EBV, *INF*γ downregulated in presence of HSV1, and *IL18R1* upregulated and *IL15* downregulated when associated with HHV6.

Applying the same criteria to TTV viral load above 10,000 copies/ml identified immunological markers that were distinct from those associated with herpesviruses, such as a decrease in T lymphocytes, notably CD4+ lymphocytes. A decrease in *IL7R* mRNA expression was also observed, as with herpesvirus. In addition, a comparison of herpes viraemia with unique TTVh detection and co-detection of TTVh and herpesvirus identified *IL15* and *CTLA4* as markers specifically associated with TTVh (data not shown). Interestingly, *IL15* expression was increased in ICU patients in the presence of TTV alone and decreased when TTV and herpesviruses were both present. Of note, an increase in *IFN*γ transcript, higher NK cells count, and a lower percentage of regulatory T cell appeared significantly associated with a low TTV viraemia (above the LOD) during the first week but not with high TTV viraemia (>10,000 copies/ml) (data not shown).

Taken together, these results suggest that the herpesvirus viraemia is associated with a prolonged exacerbated host response with concurrent immunosuppression and inflammation. This is consistent with the observed associations with severity scores and poor outcomes, notably IAI. High TTV viral load appeared associated with T-cell compartment alteration.

### Association of Individual Viruses With ICU Admission Aetiology

The percentage of patients presenting single- or multiple-positive herpesvirus viraemia (**Table 4** and **Supplementary Figure S3**) was higher in sepsis than in other groups at the end of the first week, reaching 33% for EBV, 13% for HHV6, 12% for CMV, and 12% for HSV1 in the sepsis group. This distribution was also observed at the end of the first month, except for an increase in the percentage of burns patients exhibiting herpesviruses DNAemia (from 25% for EBV to 38% for HSV1) (**Supplementary Figure S3**). The presence of EBV and CMV appeared to be the most significant contributors of this observation in sepsis patients (**Table 4**).

**Table 4:** Comparative plasma DNAemia during the first week following admission in sepsis patients *versus* a group consisting of trauma and surgery and burns patients.

Virus	Healthy^a^ (n = 175)	Sepsis^a^ (n = 107)	Other critically ill condition (n = 270)				p-value Fisher test^b^
			Trauma^a^ (n = 137)	Burns^a^ (n = 24)	Surgery^a^ (n = 109)	All CI ns^ac^ (n = 270)	
CMV	0	12	1	0	4	2	<0.0001*
EBV	1	33	7	0	7	6	<0.0001*
HHV6	2	13	4	12	8	6	0.0378
HSV1	0	12	4	4	1	3	0.005
TTVh	5	14	10	21	17	14	1

a Percentage of healthy volunteers and patients presenting single-or multiple-positive herpes (above the LOD) and/or TTVh (above 10,000 copies/ml) viral DNAemia during the first 7 days following admission in the ICU.

b p-value obtained by comparing the number of patients with positive and negative DNAemia in sepsis group versus a group consisting of trauma, burns, and surgery patients; as sepsis group and other group are frequently unbalanced in term of numbers, we only considered p values significant (*) when <0.0001.

^b^All CI ns stated for critically ill non-sepsis condition (trauma + burns + surgery).

To evaluate whether the primary infection in sepsis was responsible for this difference, we compared the viraemia detection status of sepsis patients before their first IAI (only the original pathogen is present) and the viraemia detection status of trauma, surgery, and burns patients after their first IAI (**Supplementary Table S3**). There was significantly more CMV (p = 0.027) and EBV (p < 0.001) detected in the first week in patients with sepsis compared with other conditions post IAI. Thus, infection per se does not appear to be the main driver of this difference. We thus compared the occurrence of viraemia in sepsis patients according to community-acquired primary infection *versus* hospital-acquired primary infection. Although all herpesviruses were most frequently present in association with primary hospital-acquired infection, only CMV demonstrated a significant association (p = 0.01, **Supplementary Table S4**). Conversely, high TTV viral titres tended to be mostly observed in association with community-acquired infection (**Supplementary Table S4**). Although numbers are small, it is also worth noting that the percentage of patients presenting with high TTV viraemia during the first week appeared higher in burn patients compared to other pathological conditions (**Table 4**), perhaps suggesting early preferential TTV modulation in burn patients.

Because EBV and CMV (i) are known to encode a viral homologue of IL10 and because we observed that (ii) serum IL10 level was highly correlated with EBV viraemia in the whole cohort since the first week in ICU, it was hypothesised that cellular IL10 may contribute to the specific EBV and CMV behaviour in sepsis as compared to other pathologies. Serum IL10 level was systematically higher from D1 to D28 in sepsis than in other groups of patients without IAI, regardless of the primary infection type (**Supplementary Figure S4**). Conversely, for patients that will present an IAI, IL10 level was higher from D1 to D7 in sepsis with a hospital-acquired primary infection than in community-acquired infection or other pathologies (**Supplementary Figure S4**). Of note, there were no differences in IL10 mRNA expression in blood cells in trauma-surgery-burns patients (**Supplementary Figure S4**). Overall, among those patients who will have an IAI and were primarily infected in the hospital and had high IL10 value, 75% presented with EBV or CMV DNAemia and no HSV1 or HHV6 DNAemia, whilst among those infected in the community and had low IL10 values, 38% presented with EBV or CMV DNAemia and 25% HSV1 or HHV6 DNAemia. This indicates preferential EBV/CMV reactivation in patients with primary HAI and increased IL10.

## Discussion

We previously developed a standardised semi-automated quantitative process to measure viraemia from four herpesviruses and TTV within the first week of admission in a pilot study based on a cohort of 98 patients with septic shock. The absence of association between herpesvirus viraemia and secondary infections and the observation that patients with Herpesviridae-related viraemia were more severely unwell led us to conclude that such a small cohort may not capture the diversity of patients, and additionally, focussing only on the first week may not be sufficient (10). Hence, we proposed that such quantitative tools should allow further definition of several thresholds to better discriminate between (1) non-significant viral load, (2) viral “reactivation” as a marker of immunosuppression, and (3) high viral loads supporting a true viral infection requiring treatment. This could be achieved using the larger REALISM cohort of 377 patients for which the immune status was objectively defined during the whole ICU stay.

**Figure 3:**
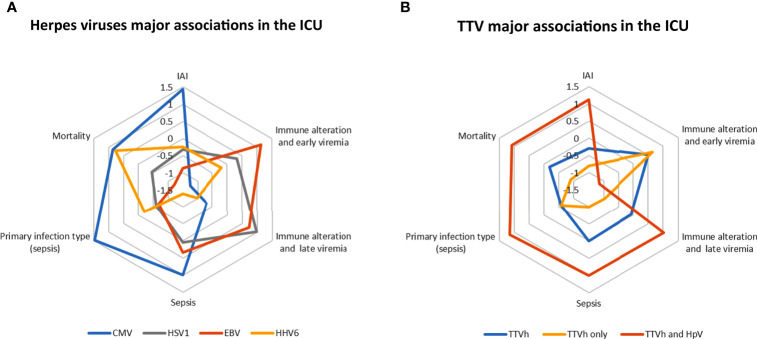
A simplified view of CMV, HSV1, EBV, HHV6, and TTV major associations in the ICU. **(A)** Comparative view of herpesviruses major associations in the ICU with the presence of CMV, HSV1, EBV, and HHV6 viruses over the month defined according to the LOD. **(B)** Comparative view of TTV major associations in the ICU with the presence of viruses over the first week categorized as high viral titre (TTVh), co-reactivation (TTV and HpV), or exclusive detection (TTVh only). IAI index (Intensive Care Unit-acquired infection): ratio of the percentages of patients with a detected viraemia according to the presence *versus* the absence of IAI during the month (source: **Table 3**). Immune alteration indexes: extent of the [**(A)** first week, **(B)** month] immune alteration associated with the presence of a virus during the week (early viraemia) and during the month (late viraemia) represented by the number of events of significantly modulated immunological and molecular markers (source: **Figure 2** and **Supplementary Figure S2**). Sepsis index: ratio of the percentages of patients with a detected viraemia during the first week with sepsis *versus* other conditions in the ICU (trauma and surgery and burns patients) (source: **Table 4**). Primary infection type index: ratio of the percentages of sepsis patients (without IAI during the month) with a detected viraemia during the month according to hospital acquired primary infection *versus* community acquired primary infection (source: **Supplementary Table S4**). Mortality index: ratio of the percentages of patients with a detected viraemia during the first week according to death at 28 days *versus* no death at 28 days (source: **Table 3**). All index ratios were harmonized in centred and scaled variables to draw the radar plot.

For the herpesviruses, we were unable to discriminate between non-significant viral load, viral reactivation, and high viral load, mainly because we observed low-level viraemia associated with sporadic viral blips. However, in the present study, we identified that virus expression depended on the extent of alteration of the immune response and on the aetiology of the pathology leading to ICU admission. Herpesvirus reactivation in the ICU was associated with IAI and a lasting exacerbated host response in all ICU patients. However, not all the herpesviruses behaved in the same way, as depicted graphically in **Figure 3A**. For example, first week EBV and HSV1 DNAemia were the most strongly associated with an early immune alteration. Moreover, sepsis patients admitted to ICUs had pre-existing medical conditions predisposing to herpesvirus viral reactivations, notably EBV and CMV, with hospital-acquired sepsis more prone to herpes viral reactivation than community-acquired sepsis. For TTV, we were able to discriminate between non-significant viral load and high viral load, hence identifying a threshold of TTV expression associated with pathophysiological features in ICU patients. Notably, high TTV viral load together with herpesvirus reactivation in the ICU was associated with IAI from the first week of admission, earlier than herpesvirus alone, as depicted graphically in **Figure 3B**. Additionally, high early TTV viraemia was associated with immune alteration.

Single viraemia was more frequent than viraemia from multiple viruses, as previously observed using metagenomic sequencing (16), and the percentage of ICU patients who had at least one Herpesviridae-related viraemia increased over time, as noted by others (9, 13). Our study adds to the literature evaluating herpesviruses reactivation in patients with critically ill conditions including sepsis summarised in **Table 5**. The prevalence of herpesviruses in the plasma from ICU patients is roughly parallel to their level of prevalence in the healthy population (34, 35). Detection rates overall are in the same range of what was previously observed in ICU patients (15, 36, 37), essentially lower than in immunosuppressed transplant patients (38). Nevertheless, sepsis patients seemed more prone to viral reactivation than patients presenting other pathologies (9, 11, 13). EBV and TTV were the viruses most frequently co-detected, although each virus was predominantly detected alone; this seems to exclude any helper effect of EBV infection in TTV replication in these ICU/immune-compromised populations, as previously described in multiple sclerosis (39). In the REALISM ICU cohort, the TTV prevalence ranged from 51% in healthy control to 58% in septic patients, slightly less than the prevalence observed in another large ICU cohort screened with the same PCR tool, ranging from 60% in healthy control patients to 77% in septic patients (9). Taking into account a high TTV viral load (>10,000 copies/ml), the prevalence is three times higher in ICU patients (15%) than in healthy volunteers (5%), peaking at 21% for burns patients. Of note are the high TTV viral titres in ICU patients, which appeared several log lower than in transplanted patients (40).

**Table 5:** Cumulative percentages of reactivation of various herpesviruses and TTV in healthy individuals, critically ill patients during ICU stay, and immunosuppressed transplant patients.

Overall conditions	References	Description of cohorts	CMV	EBV	HHV6	HSV1	TTV
Healthy individuals	This study	ICU age-matched patients (n = 175)	0	1	2	0	51
	(9)	ICU age-matched patients (n = 175)	0	1	4	0	60
	(34)	Blood virome RNA seq. (n = 8240)	<1%*	14*	5*	<1%*	
	(35)	Tissue virome DNA seq. (n = 547)	8*	39*	6*	6*	
ICU patients	This study	ICU all patients (n = 377)	9	18	10	12	
	(15)	Med./anaesth. ICU (blood, urine) (n = 60)	8*	23*			
	(36 ^a^)	ICU patients	14–40				
	(37)	ICU patients (n = 1,556)				12	
Septic patients	This study	Sepsis (n = 107)	15	36	14	19	58
	(9)	Sepsis (n = 235)	22	32	13	18	77
	(13)	Septic shock patients (n = 329)	18	48	24	26	
	(11)	Septic paediatric patients (n = 73)	5	44	8	4	89
Critically ill non-septic patients	This study	Trauma, burn, surgery (n = 270)	7	10	9	10	57
	(9)	Surgical/medical ICU (n = 55)	0	5	0	0	64
Transplanted patients	(38 ^b^)	Immunosuppressed transplant patients (blood)	13*	44*	32*	3*	

Presented reactivations were determined by PCR from plasma samples. Data obtained by other method or using a different biological compartment are indicated by an asterisk.

a Review article describing 26 cohorts.

b Study analysing 16,069 PCR assays undertaken on blood obtained from immunosuppressed patients.

The association between several herpesviruses and worse outcomes has been widely observed in the literature (14, 16, 41, 42). We observed a similar association with patients that had at least one herpesvirus viraemia during the month: these patients were more severely unwell, with worse outcomes such as mortality and the occurrence of ICU acquired infection. The presence of CMV and HHV6 from the first week in ICU appeared to correlate with a mortality at D28. However, EBV DNAemia was not associated with mortality in contrast to previous observations (9, 13), whilst the mortality rate in HSV1 patients was not increased compared to the overall mortality rate in the ICU as has been noted previously (18, 37). We also observed that patients with multiple herpesvirus viraemic events had higher mortality compared to those with a single viraemic event as previously described (10, 13), but high load TTV together with at least one herpesvirus appeared to be associated with a lower mortality, as suggested in septic shock patients (10). The presence of each individual herpesvirus, i.e., CMV, EBV, HHV6, and HSV1, during the month is significantly associated with the occurrence of IAI. This extends the observations that (i) patients under mechanical ventilation with active CMV infection were more prone to developing bacterial nosocomial infections (1, 43) and that (ii) septic patients who had detectable HSV in blood had increased risk of developing opportunistic bacterial infections (9). Of note, we previously observed a trend between EBV detection in the first week of admission and IAI (10), and others observed an association with fungal infections (9) in sepsis patients. Patients with multiple viraemic events, including those with TTV viraemia, had higher IAI (44%) compared to those with single or no viraemic events (22% and 19%, respectively). Additionally, high TTV viral load together with herpesvirus co-detection during the first week was significantly associated with the occurrence of IAI events during the month. Whilst the reason for this association is not clear, it can be speculated that individuals with multiple viral reactivations were more immunosuppressed and hence at greater risk of developing IAI.

Our results demonstrate that ICU patients with herpesvirus viraemia maintain an exacerbated inflammatory profile (IL6, *C3AR1*, *IL18R1*, and *HP*) together with anti-inflammatory characteristics (*IL1R2* and IL10) and deeply altered immunological synapses (*CD74*, *CD3D*, *CD40L*, and *TNFSF4/OX40L*, including *BTLA* and *ICOS* for EBV) and contributors to differentiation (*STAT4* and *IL7R*) and severity marker (*CX3CR1*, retrotransposons), consistent with the observed associations with severity scores and poor outcomes. It is worth noting that individuals who went on to develop EBV reactivation appeared to present with more severe immune alteration. Consistent with these results, it was recently shown that EBV reactivation in sepsis was correlated with the SRS1 immunocompromised sepsis transcriptomic endotype (16). During the first week, TTV DNAemia appeared specifically associated with modulation of the lymphoid compartment, including decreased T cells and high expression of *IL15* associated with high TTV titre. Additionally, regulatory T-cell decrease, NK-cell increase, and high expression of *IFN*γ associated with TTV presence (75% of patients without high TTV viral load) without any herpesvirus. Such a transient increase in *IFN*γ transcription in blood of ICU patients is different from the absence of TTV-induced *IFN*γ production observed in many tissues of healthy individuals (35). However, the dynamics of the immune response *versus* the TTV viral load may follow a distinct course depending on the context, as exemplified by the dynamics over several months described in haematopoietic stem-cell-transplanted patients (40).

There is no consensus definition of viral reactivation in critically ill patients, and whilst many authors report data on viraemia (blood compartment), others include viral detection in the respiratory tract (36, 44). Beyond differences due to viral replication strategies (45), it is unclear what factors may account for differences in viral reactivation. The higher serum IL10 level in sepsis compared to all other ICU pathologies may suggest that EBV and CMV reactivation could be triggered by molecular events mediated by stress, local trauma, or unrelated infections (45, 46), rather than associated with pre-existing immune suppression (11, 45, 47). Alternatively, we observed low-level viraemia or viral “blips” for herpesviruses rather than significant viral rebound; hence, reactivation may result from increased inflammation in sanctuary sites or reservoirs as described in HIV infection (48, 49) or to immunosurveillance activities of neighbouring cells (50). The changes observed in TTV viraemia in ICU patients in this study indicate the need for further exploration of the process of reactivation/modulation of expression of TTV. This may in turn help to further decipher the as-yet-unknown cellular source of TTV replication (51) and thus lead to a better understanding of whether TTV, a major component of the human virome, impacts the development of our immune system (52).

Our study has some limitations. First, patient recruitment from a single centre could limit the generalisability of our results to other centres. However, this approach was chosen to ensure standardisation and reproducibility of the measurement of viraemia and immune parameters, together with relative homogeneity in patient management. Second, the absence of IgG and IgM serology for each herpesvirus did not allow us to systematically separate reactivation from primary infection, although we also had to consider that transfusion or immunoglobulin infusion could lead to passive transfer of IgG. Additionally, detection of virions was performed using plasma samples only. Detection of viruses using supplementary specimen types such as whole blood, urine, and oropharyngeal and broncho-alveolar lavage fluids may provide additional information, especially when considering the risk of catheter-associated urinary tract infection and ventilator-associated pneumonia in ICU patients and the potential for local viral reactivation in organs (35, 42, 44). Finally, the follow-up of patients for only 1 month may be insufficient to identify the long-term characteristics and consequences of sporadic herpesvirus viraemia and “slow-and-low” TTV course. Nevertheless, this study has demonstrated that there may be utility in further evaluating EBV detection and TTV quantitation during the follow-up of sepsis patients to help guide clinical management, as this may potentially drive the development of therapies that target the immune system. Furthermore, there may be benefits from designing clinical trials to understand EBV detection and TTV quantitation and dynamics over longer periods including after ICU discharge in order to tackle the challenges in the management of post-sepsis syndrome (6).

## Conclusion

In conclusion, by applying a semi-automated process of viral DNAemia determination and an assessment of the host response, our study demonstrated that reactivation of herpesviruses depended on the pathology that led to ICU admission and was associated with the extent of alteration of the immune response. Regulation of TTV viraemia appeared specifically associated with modulation of the lymphoid compartment of the immune system. Thus, comparisons of different pathologies leading to ICU admission are useful models to better understand mechanisms controlling the emergence of herpesviruses and TTV.

## Data Availability Statement

All the data related to the study are available upon motivated request to the corresponding author. Sharing of the data is submitted to the approval of the REALISM consortium steering committee as per consortium agreement.

## Ethics Statement

The studies involving human participants were reviewed and approved by the institutional ethical review board (Comité de Protection des Personnes Sud-Est II) under number 2015-42-2. This clinical study was also registered at clinicaltrials.gov (NCT02638779). The patients/participants provided their written informed consent to participate in this study.

## REALISM Study Group

HCL: Sophie Arnal, Caroline Augris-Mathieu, Frédérique Bayle, Liana Caruso, Charles-Eric Ber, Asma Ben-Amor, Anne-Sophie Bellocq, Farida Benatir, Anne Bertin-Maghit, Marc Bertin-Maghit, André Boibieux, Yves Bouffard, Jean-Christophe Cejka, Valérie Cerro, Jullien Crozon-Clauzel, Julien Davidson, Sophie Debord-Peguet, Benjamin Delwarde, Robert Deleat-Besson, Claire Delsuc, Bertrand Devigne, Laure Fayolle-Pivot, Alexandre Faure, Bernard Floccard, Julie Gatel, Charline Genin, Thibaut Girardot, Arnaud Gregoire, Baptiste Hengy, Laetitia Huriaux, Catherine Jadaud, Alain Lepape, Véronique Leray, Anne-Claire Lukaszewicz, Guillaume Marcotte, Olivier Martin, Marie Matray, Delphine Maucort-Boulch, Pascal Meuret, Céline Monard, Florent Moriceau, Guillaume Monneret, Nathalie Panel, Najia Rahali, Thomas Rimmele, Cyrille Truc, Thomas Uberti, Hélène Vallin, Fabienne Venet, Sylvie Tissot, Abbès Zadam

bioMérieux: Sophie Blein, Karen Brengel-Pesce, Elisabeth Cerrato, Valérie Cheynet, Emmanuelle Gallet-Gorius, Audrey Guichard, Camille Jourdan, Natacha Koenig, François Mallet, Boris Meunier, Virginie Moucadel, Marine Mommert, Guy Oriol, Alexandre Pachot, Estelle Peronnet, Claire Schrevel, Olivier Tabone, Julien Textoris, Javier Yugueros Marcos

BIOASTER: Jérémie Becker, Frédéric Bequet, Yacine Bounab, Florian Brajon, Bertrand Canard, Muriel Collus, Nathalie Garcon, Irène Gorse, Cyril Guyard, Fabien Lavocat, Philippe Leissner, Karen Louis, Maxime Mistretta, Jeanne Moriniere, Yoann Mouscaz, Laura Noailles, Magali Perret, Frédéric Reynier, Cindy Riffaud, Mary-Luz Rol, Nicolas Sapay, Trang Tran, Christophe Vedrine

SANOFI: Christophe Carre, Pierre Cortez, Aymeric De Monfort, Karine Florin, Laurent Fraisse, Isabelle Fugier, Sandrine Payrard, Annick Peleraux, Laurence Quemeneur

ESPCI: Andrew Griffiths, Stephanie Toetsch

GSK: Teri Ashton, Peter J. Gough, Scott B. Berger, David Gardiner, Iain Gillespie, Aidan Macnamara, Aparna Raychaudhuri, Rob Smylie, Lionel Tan, Craig Tipple.

## Author Contributions

FM, FR, PL, LQ, AG, VM, AP, FV, GM, AP, TR, and JT made substantial contributions to conception and design. MP, FR, and FV contributed to the acquisition of data. FM, LD, and BM made substantial contributions to the analysis, interpretation, and drafting of the manuscript. VM, LT, KB-P, and JT have been involved in the critical revision of the manuscript. All authors read and approved the final manuscript, and agreed to be accountable for all aspects of the work in ensuring that questions related to the accuracy or integrity of any part of the work are appropriately investigated and resolved.

## Funding

The project was funded by a consortium: bioMérieux, SANOFI, GlaxoSmithKline, Ecole Supérieure de Physique Chimie Industrielles de la Ville the Paris–PSL Research University, the University Hospital Hospices Civils de Lyon, and the microbiology technological institute BIOASTER. The project was financially supported in part by public funding through BIOASTER and Hospices Civils de Lyon. The project will be audited annually by the French National Research Agency (“Investissement d’Avenir” program; grant no. ANR7107AIRT703). The funder was not involved in the study design, collection, analysis, interpretation of data, the writing of this article or the decision to submit it for publication.

## Conflict of Interest

FM, VM, AP, KB-P, and JT are employees of bioMérieux SA, an *in vitro* diagnostic company. LD was employed by IVIDATA. BM was employed by company Soladis Inc. FV, GM, AP, TR, and JT are employees of Hospices Civils de Lyon. FM, LD, BM, VM, AP, FV, GM, AL, TR, KB-P, and JT work in a joint research unit, co-funded by the Hospices Civils de Lyon and bioMérieux. LT is an employee of and hold stock and shares in GlaxoSmithKline. LQ is an employee of Sanofi Pasteur.

The remaining authors declare that the research was conducted in the absence of any commercial or financial relationships that could be construed as a potential conflict of interest.

## Publisher’s Note

All claims expressed in this article are solely those of the authors and do not necessarily represent those of their affiliated organizations, or those of the publisher, the editors and the reviewers. Any product that may be evaluated in this article, or claim that may be made by its manufacturer, is not guaranteed or endorsed by the publisher.

## Abbreviations

CCTIRS, Comité Consultatif sur le Traitement de l’Information en matière de Recherche en Santé; CMV, cytomegalovirus; CNIL, Commission Nationale Informatique et Liberté; CT, crossing threshold; DNA, desoxyribonucleic acid; EBV, Epstein–Barr virus; EFS, Etablissement Français du Sang; HAI, healthcare-associated infection; HHV, human herpesvirus; HSV, herpes simplex virus; IAI, ICU-acquired infection; IC, internal control; ICU, Intensive Care Unit; LOD, limit of detection; LOQ, limit of quantification; PCR, polymerase chain reaction; SOFA, Sequential Organ Failure Assessment score; TTV, Torque teno virus.
