# Supplementary material for: Herpes DNAemia and TTV Viraemia in Intensive Care Unit Critically Ill Patients: A Single-Centre Prospective Longitudinal Study
Source: Front Immunol. 2021 Nov 2;12:698808. doi: 10.3389/fimmu.2021.698808 (PMC8593420; doi:10.3389/fimmu.2021.698808)
Supplement: Supplementary file 1 [file DataSheet_1.docx]

|  | Density = f (titre in copies/ml) | Occurrence= f(number of events) |
| --- | --- | --- |
| EBV | 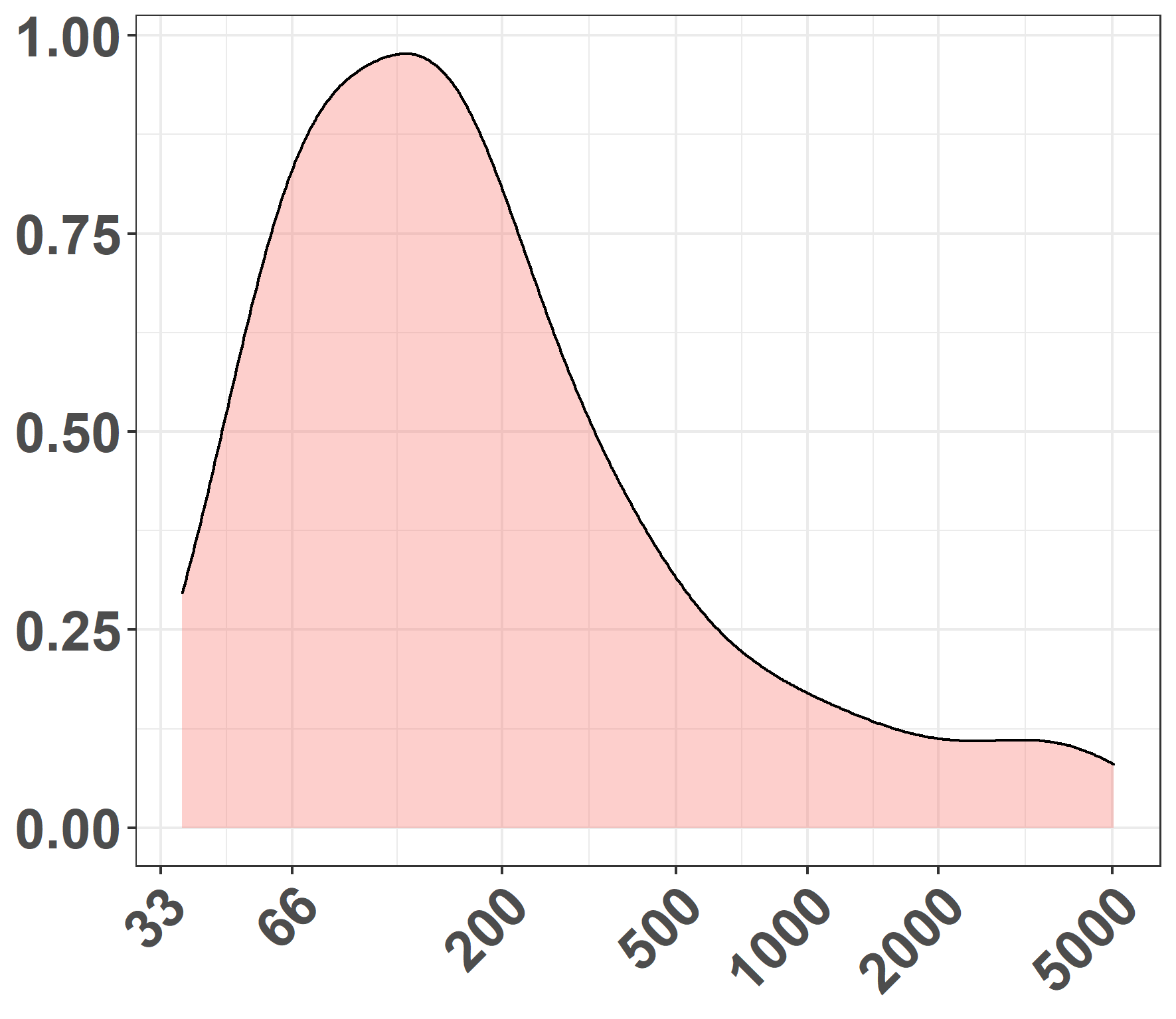 | 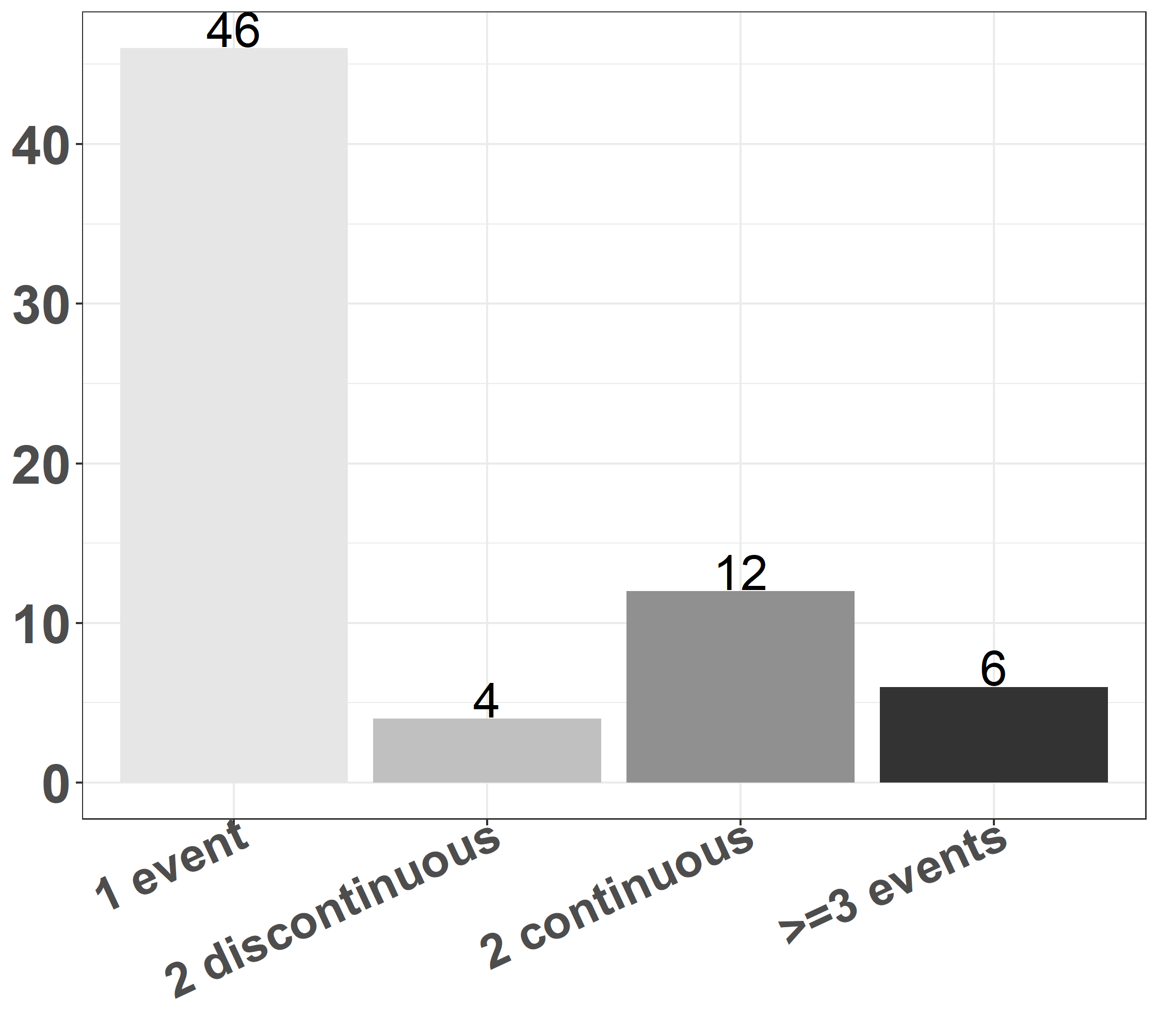 |
| CMV | 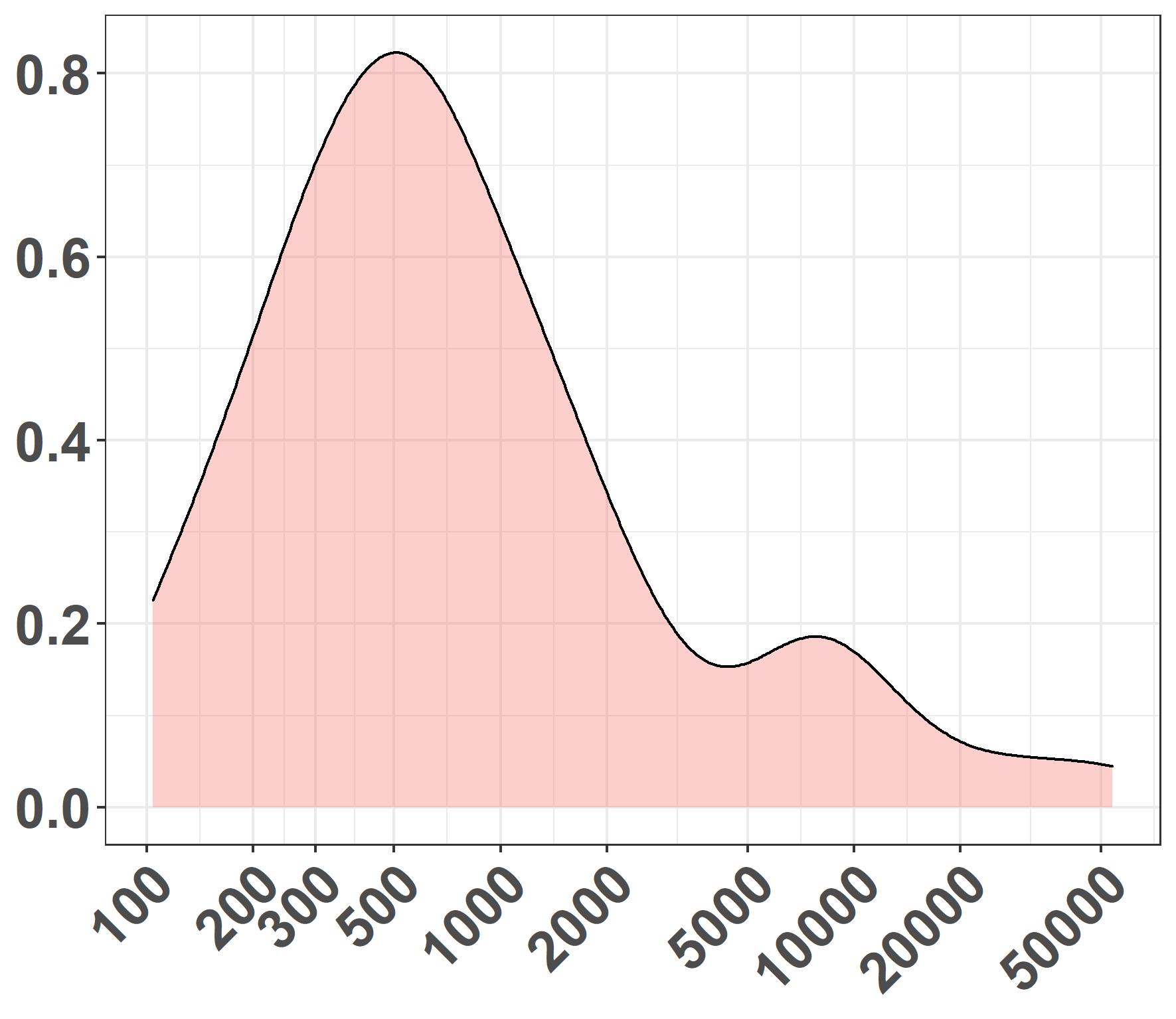 | 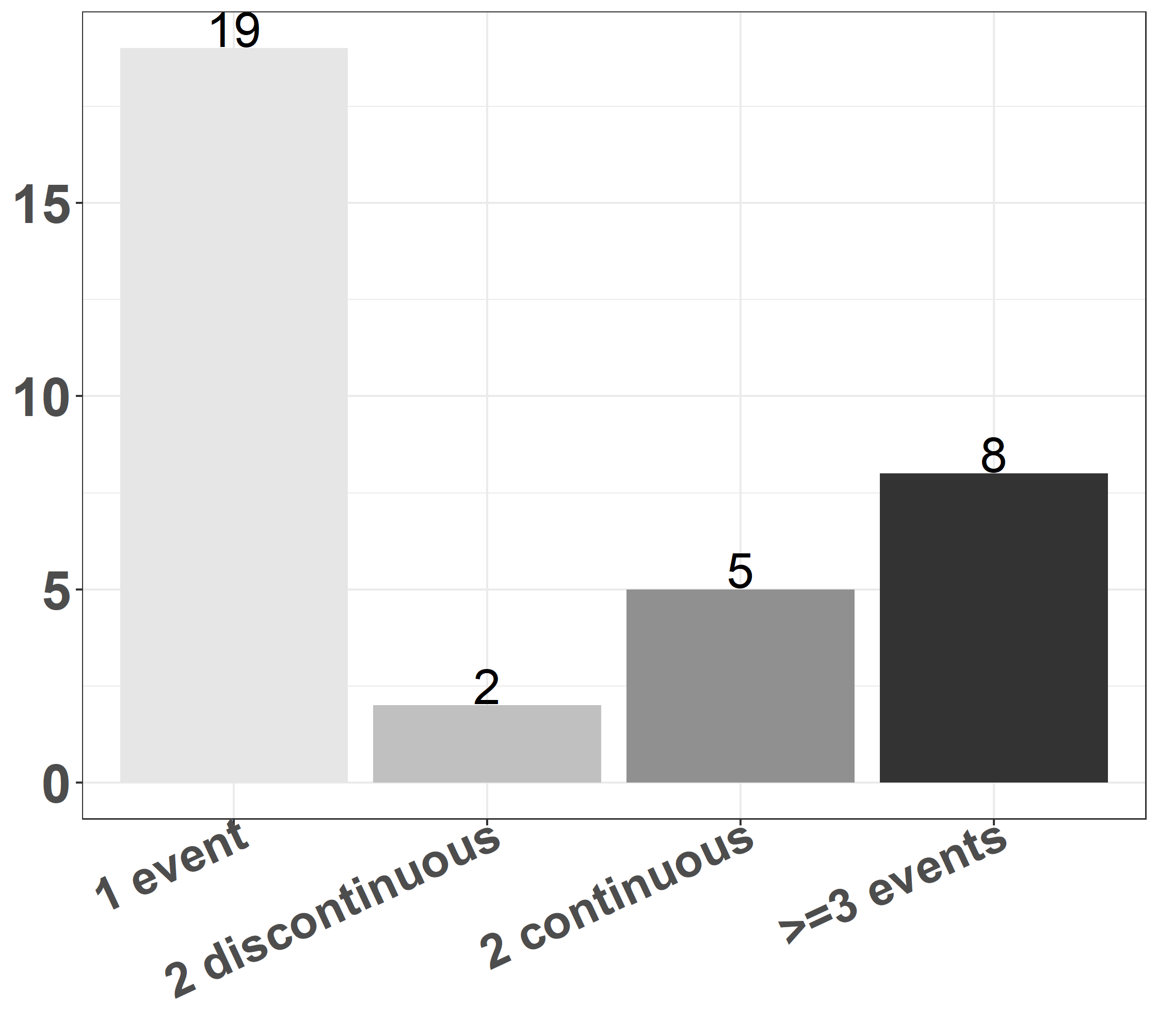 |
| HSV1 | 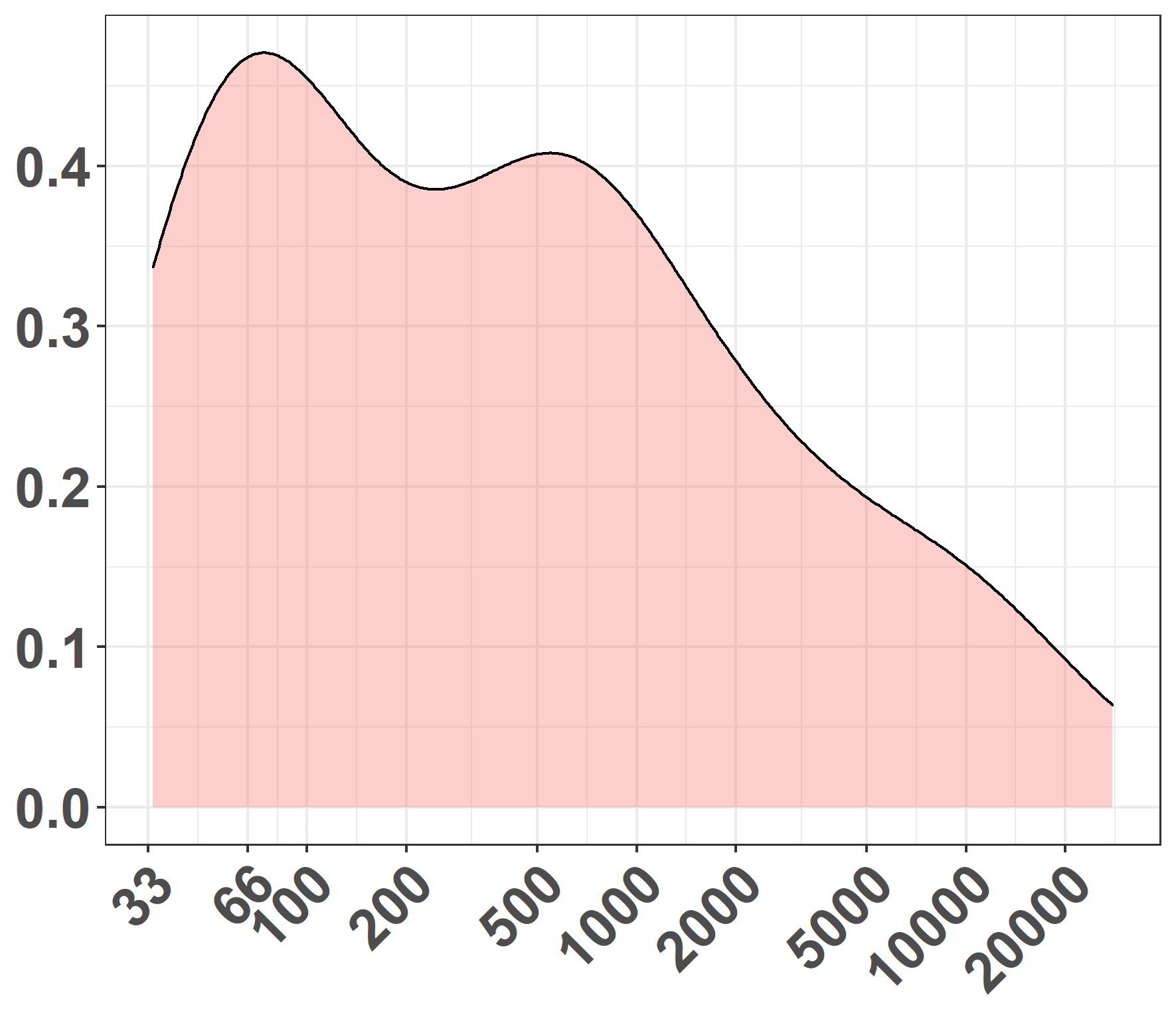 | 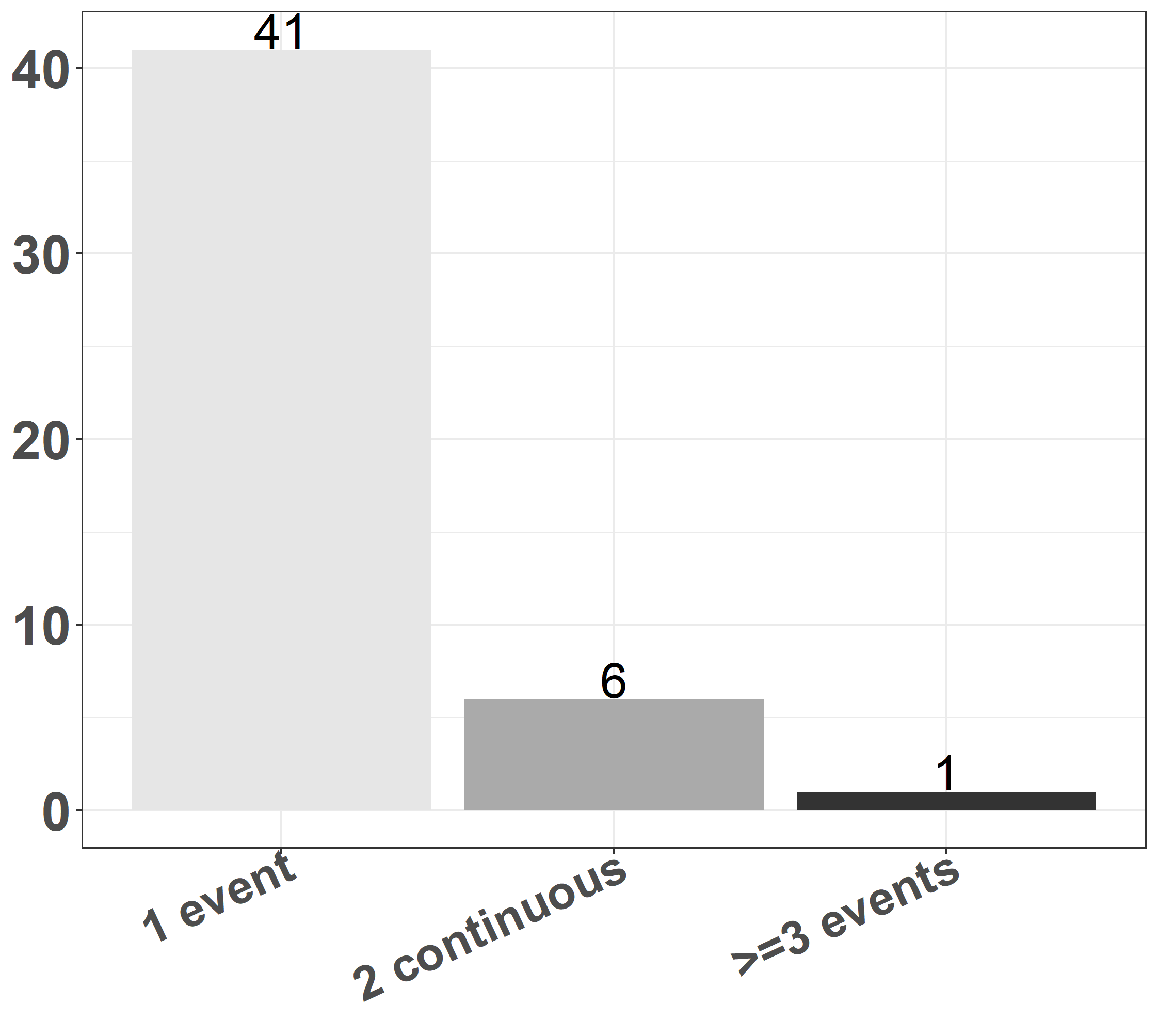 |
| HHV6 | 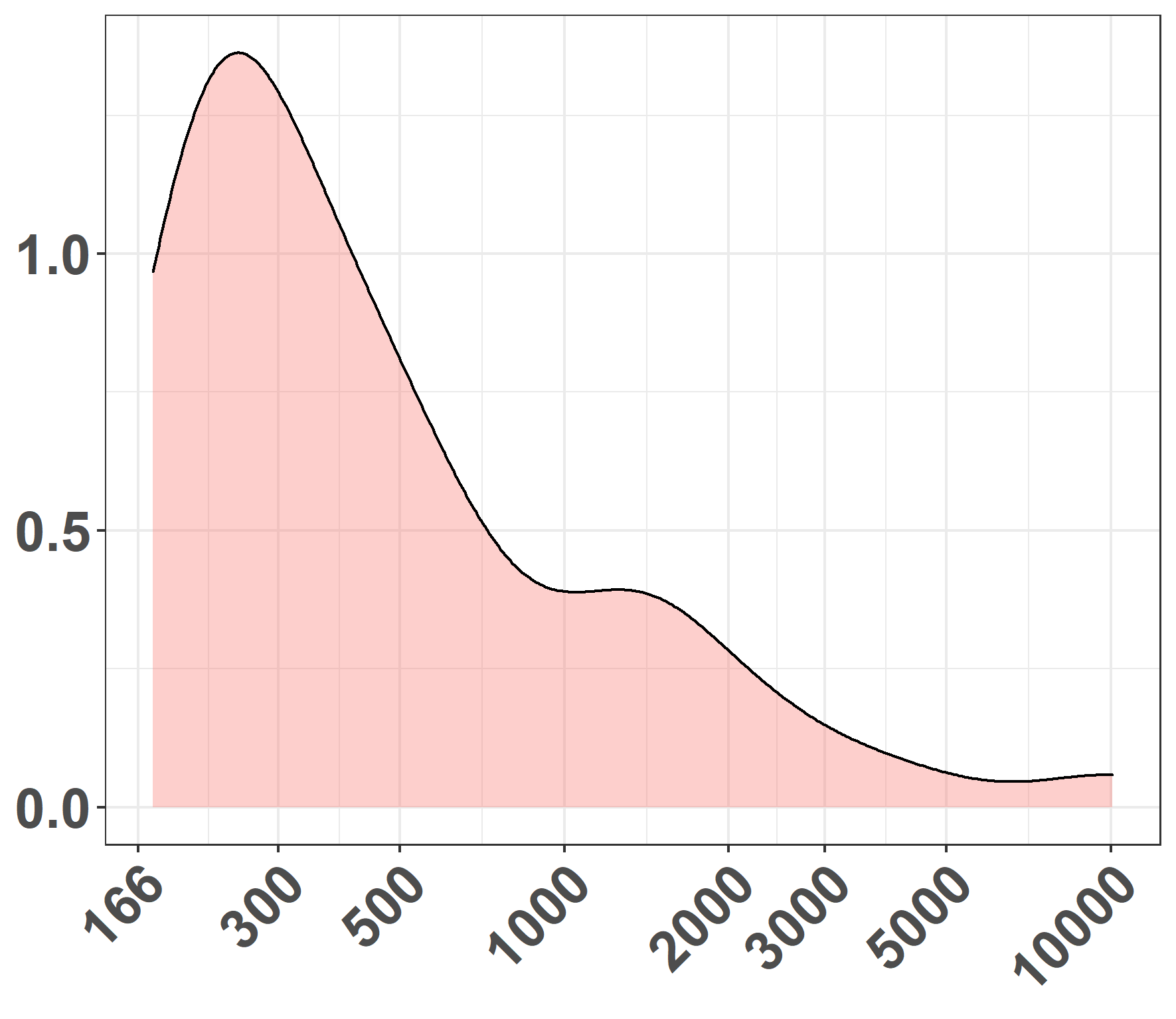 | 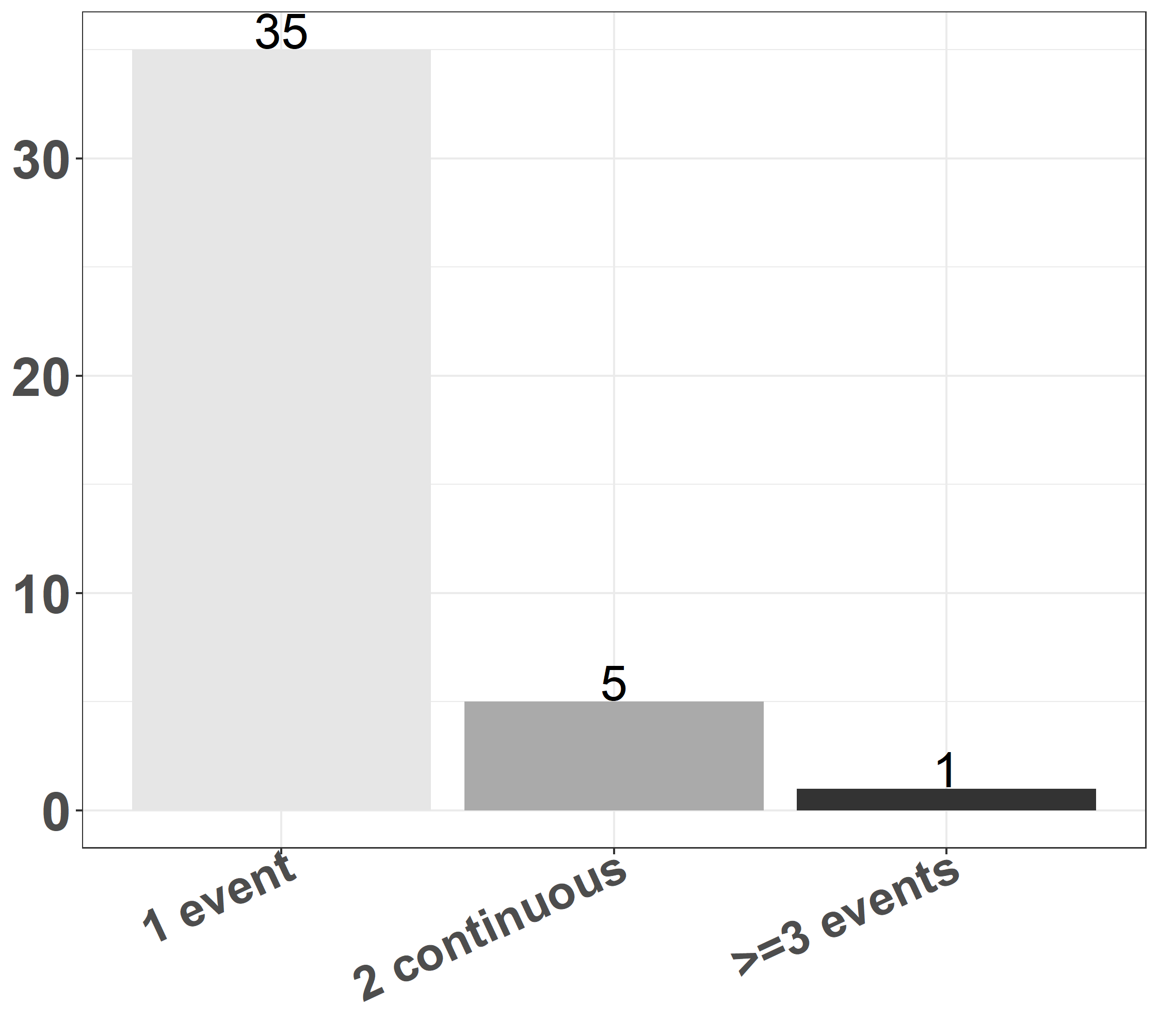 |
| TTV | 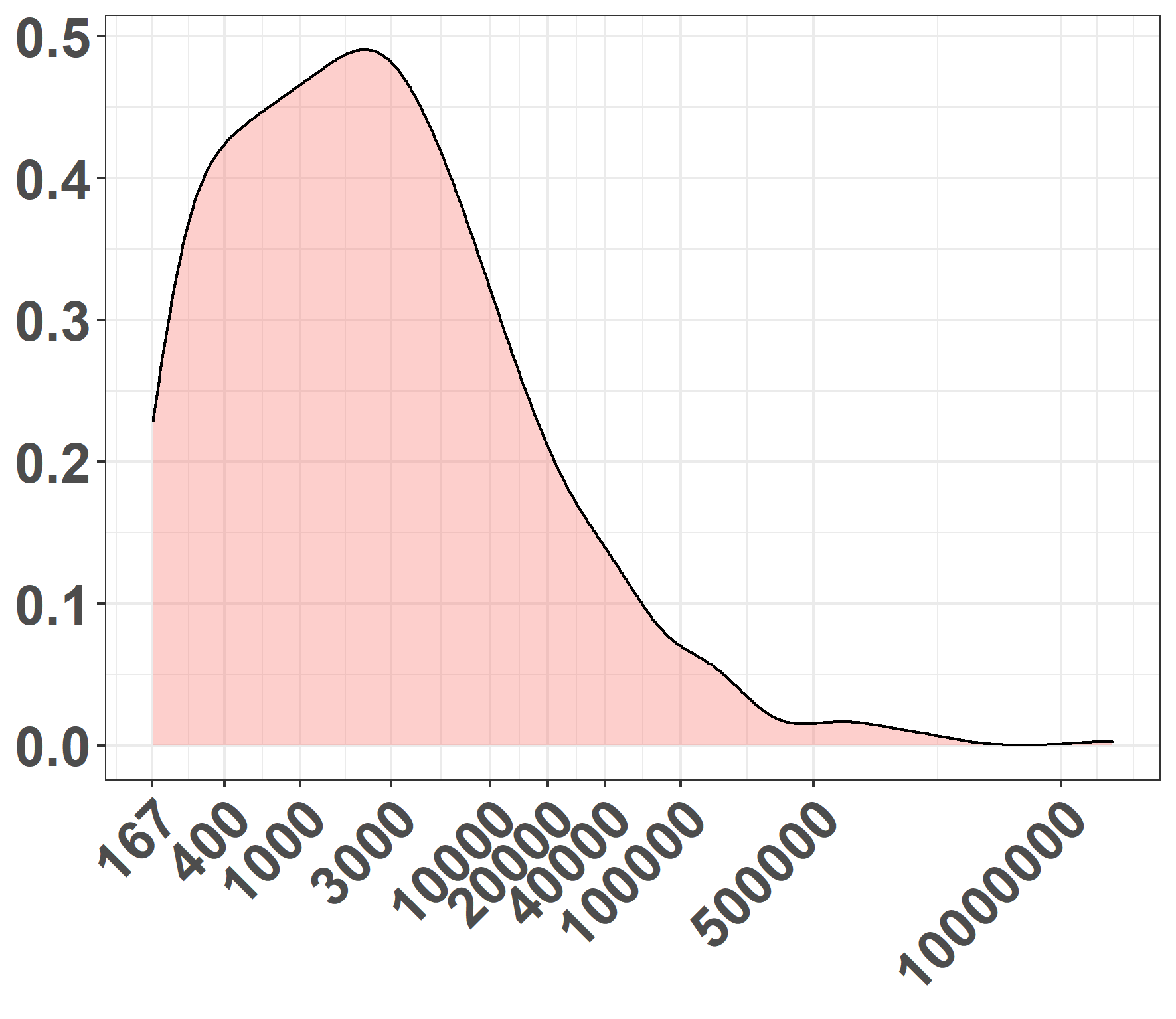 | 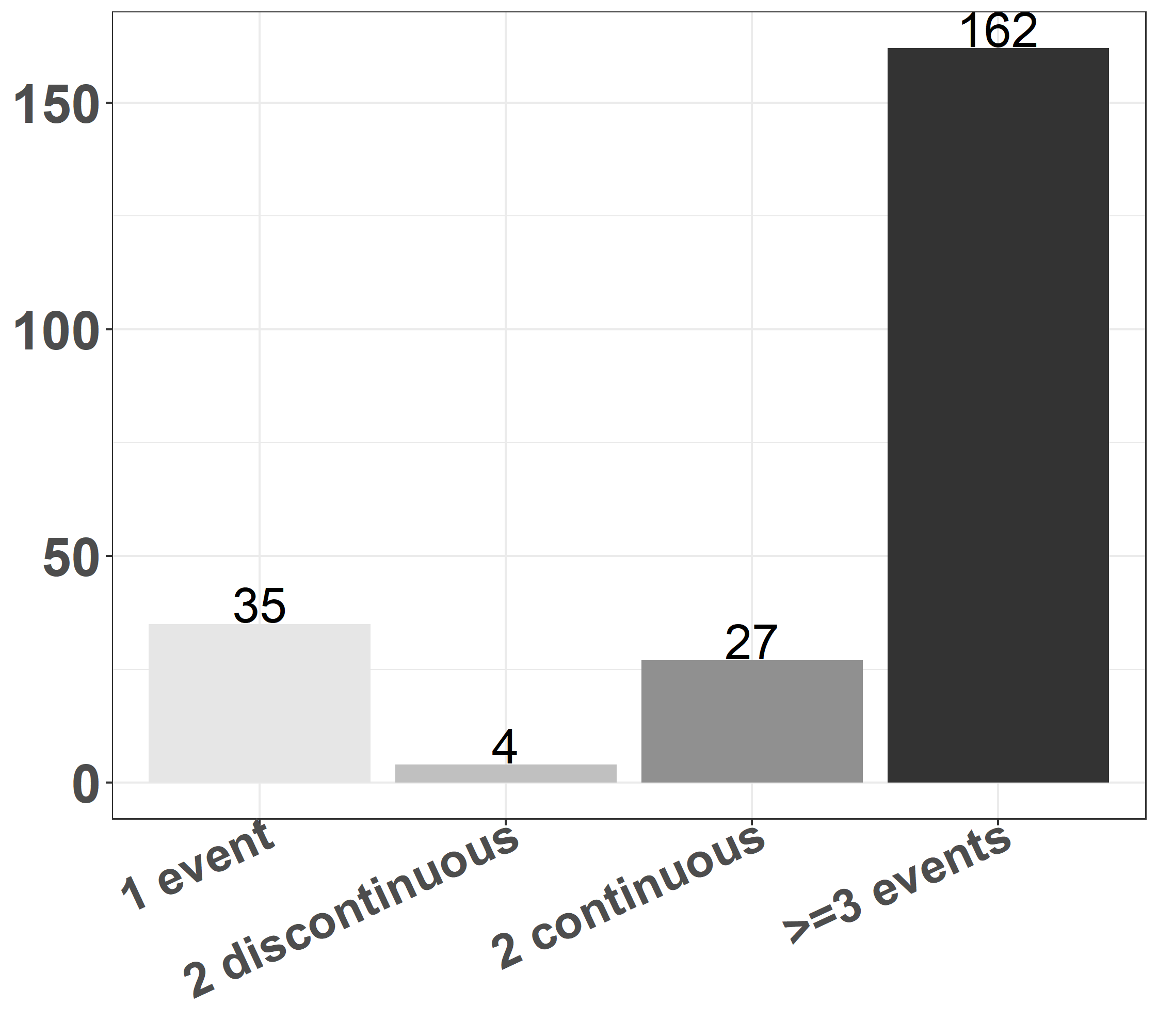 |

**Supplementary Figure 1. Distribution and occurrence of viral DNAmia**. **(A)** Distribution of the viral titres identified above the LOD for all ICU patient’s samples. **(B)** Occurence of DNAemia event classified as unique, rare (2 events) or frequent (at least 3 out 5 events during the 5 time points of collection)

**Supplementary Table 1**: ICU patient characteristics at admission and outcomes according to Herpesvirus plasma DNAemia during the first week (D1 to D7) in ICU (excluding TTV)

|  | **Viremia not present (n= 281)** | **At least one herpes viremia (n= 96)** | **Whole cohort (n = 377)** | ***p value*** |
| --- | --- | --- | --- | --- |
| **Demographics** |  |  |  |  |
| Age years | 54.8 [44-70] | 64.8 [59-75.2] | 57.4 [47-71] | <0.001*** |
| Gender (female) | 98 (35%) | 31 (32%) | 129 (34%) | 0.645 |
| **Pathologies** |  |  |  | <0.001*** |
| Sepsis | 54 (19%) | 53 (55%) | 107 (28%) |  |
| Trauma | 118 (42%) | 19 (20%) | 137 (36%) |  |
| Surgery | 88 (31%) | 21 (22%) | 109 (29%) |  |
| Burn | 21 (7%) | 3 (3%) | 24 (6%) |  |
| **Admission** |  |  |  |  |
| SAPSII day 1 | 29 [18-38] | 41.4 [29-52.2] | 32.1 [20-44] | <0.001*** |
| SOFA score day 1 | 4.4 [1-8] | 7 [4-10] | 5.1 [1-8] | <0.001*** |
| Charlson score day 1 | 1.3 [0-2] | 2.1 [0-3] | 1.5 [0-2] | <0.001*** |
| **Sepsis primary infection type** | | | | 0.016* |
| Community acquired | 44 (16%) | 32 (33%) | 76 (20%) |  |
| Hospital acquired | 10 (4%) | 21 (22%) | 31 (8%) |  |
| **Sepsis primary infection site** | | | | 0.394 |
| Abdominal | 21 (7%) | 21 (22%) | 42 (11%) |  |
| Pulmonary | 11 (4%) | 16 (17%) | 27 (7%) |  |
| Others | 22 (8%) | 16 (17%) | 38 (10%) |  |
| **Treatment** |  |  |  |  |
| Hydrocortisone | 20 (7%) | 21 (22%) | 41 (11%) | <0.001*** |
| Vasopressors day 1 | 129 (46%) | 68 (71%) | 197 (52%) | <0.001*** |
| **Chemistry and hematology at admission** | | | | |
| Monocytes (G/L) | 1.1 [0.7-1.3] | 1 [0.6-1.3] | 1.1 [0.7-1.3] | 0.055 |
| Lymphocytes (G/L) | 1.4 [0.8-1.8] | 1.4 [0.8-2] | 1.4 [0.8-1.8] | 0.738 |
| Neutrophils (G/L) | 12.3 [8.4-14.9] | 13.4 [7.5-16.6] | 12.6 [8-15.1] | 0.926 |
| Lactate concentration (mM) | 2.6 [1.7-3.2] | 3.3 [1.7-3.7] | 2.8 [1.7-3.4] | 0.189 |
| **Outcomes** |  |  |  |  |
| Hemodialysis duration (days) | 15.9 [2-9.8] | 11.1 [1.2-20.5] | 13.2 [1.8-14.5] | 0.939 |
| Mechanical Ventilation (days) | 10.3 [1-9] | 6.2 [1-7] | 8.8 [1-9] | 0.193 |
| ICU length of stay (days) | 11 [3-11] | 13 [5-15] | 11.6 [4-12.2] | 0.002** |
| Hospital length of stay (days) | 20.6 [9-26] | 24.8 [12-31] | 21.6 [9-27] | 0.036* |
| ICU Mortality D28 | 8 (3%) | 13 (14%) | 21 (6%) | <0.001*** |
| At least one IAI D28 | 69 (25%) | 28 (29%) | 97 (26%) | 0.372 |

Categorical variables are expressed as n(%) and continuous variables as median [Q1-Q3]. Comparisons between detected or not detected Herpes viruses (CMV, EBV, HHV6, HSV1), were performed with a Chi−squared test for qualitative variables and Wilcoxon test for quantitative variables, as appropriate. *P* values with stars indications represent significance at p < 0.05. IAI ICU acquired infection ¨ICU intensive care unit, SOFA sequential organ failure assessment, SAPS Simplified Acute Physiology Score

**Supplementary Table 2**: ICU patient characteristics at admission and outcomes according to plasma TTV DNAemia during the first month (D1 to D28) in ICU

|  | | **TTV viremia not present (n= 149)** | | **TTV viremia**  **(n= 228)** | | **Whole cohort (n = 377)** | | ***p value*** | |
| --- | --- | --- | --- | --- | --- | --- | --- | --- | --- |
| **Demographics** | |  | |  | |  | |  | |
| Age years | | 55.1 [46-68] | | 58.8 [47-73] | | 57.4 [47-71] | | 0.026* | |
| Gender (female) | | 54 (36%) | | 75 (33%) | | 129 (34%) | | 0.503 | |
| **Pathologies** | |  | |  | |  | | 0.421 | |
| Sepsis | | 43 (29%) | | 64 (28%) | | 107 (28%) | |  | |
| Trauma | | 54 (36%) | | 83 (36%) | | 137 (36%) | |  | |
| Surgery | | 39 (26%) | | 70 (31%) | | 109 (29%) | |  | |
| Burn | | 13 (9%) | | 11 (5%) | | 24 (6%) | |  | |
| **Admission** | |  | |  | |  | |  | |
| SAPSII day 1 | | 32.1 [20-44] | | 32.1 [20-43.2] | | 32.1 [20-44] | | 0.871 | |
| SOFA score day 1 | | 5.2 [2-8] | | 5 [1-8] | | 5.1 [1-8] | | 0.643 | |
| Charlson score day 1 | | 1.3 [0-2] | | 1.6 [0-3] | | 1.5 [0-2] | | 0.105 | |
| **Sepsis primary infection type** | | | | | | | | 0.814 | |
| Community acquired | | 30 (20%) | | 46 (20%) | | 76 (20%) | |  | |
| Hospital acquired | | 13 (9%) | | 18 (8%) | | 31 (8%) | |  | |
| **Sepsis primary infection site** | | | | | | | | 0.09 | |
| Abdominal | | 12 (8%) | | 30 (13%) | | 42 (11%) | |  | |
| Pulmonary | | 11 (7%) | | 16 (7%) | | 27 (7%) | |  | |
| Others | | 20 (13%) | | 18 (8%) | | 38 (10%) | |  | |
| **Treatment** | |  | |  | |  | |  | |
| Hydrocortisone | | 14 (9%) | | 27 (12%) | | 41 (11%) | | 0.456 | |
| Vasopressors day 1 | | 79 (53%) | | 118 (52%) | | 197 (52%) | | 0.81 | |
| **Chemistry and hematology at admission** | | | | | | | | | |
| Monocytes (G/L) | | 1.1 [0.7-1.3] | | 1.1 [0.6-1.4] | | 1.1 [0.7-1.3] | | 0.883 | |
| Lymphocytes (G/L) | | 1.4 [0.8-1.8] | | 1.4 [0.8-1.8] | | 1.4 [0.8-1.8] | | 0.918 | |
| Neutrophils (G/L) | | 12.5 [7.8-15.7] | | 12.6 [8.4-14.6] | | 12.6 [8-15.1] | | 0.886 | |
| Lactate concentration (mM) | | 3 [1.7-3.6] | | 2.7 [1.7-3.3] | | 2.8 [1.7-3.4] | | 0.244 | |
| **Outcomes** | |  | |  | |  | |  | |
| Hemodialysis duration (days) | | 13.3 [2-8] | | 13.1 [1.2-20.5] | | 13.2 [1.8-14.5] | | 0.62 | |
| Mechanical Ventilation days | | 10 [1-10] | | 7.9 [1-8] | | 8.8 [1-9] | | 0.396 | |
| ICU length of stay (days) | | 11.7 [4-13] | | 11.5 [4-12] | | 11.6 [4-12.2] | | 0.611 | |
| Hospital length of stay | | 22.6 [8-28.5] | | 21 [9-26] | | 21.6 [9-27] | | 0.677 | |
| ICU Mortality D28 | | 12 (8%) | | 9 (4%) | | 21 (6%) | | 0.089 | |
| At least one IAI D28 | | 37 (25%) | | 60 (26%) | | 97 (26%) | | 0.747 | |

Categorical variables are expressed as n(%) and continuous variables as median [Q1-Q3]. Comparisons between detected or not detected TTV were performed with a Chi−squared test for qualitative variables and Wilcoxon test for quantitative variables, as appropriate. *P* values with stars indications represent significance at p < 0.05. IAI ICU acquired infection ¨ICU intensive care unit, SOFA sequential organ failure assessment, SAPS Simplified Acute Physiology Score

| **A** | Cellular markers | **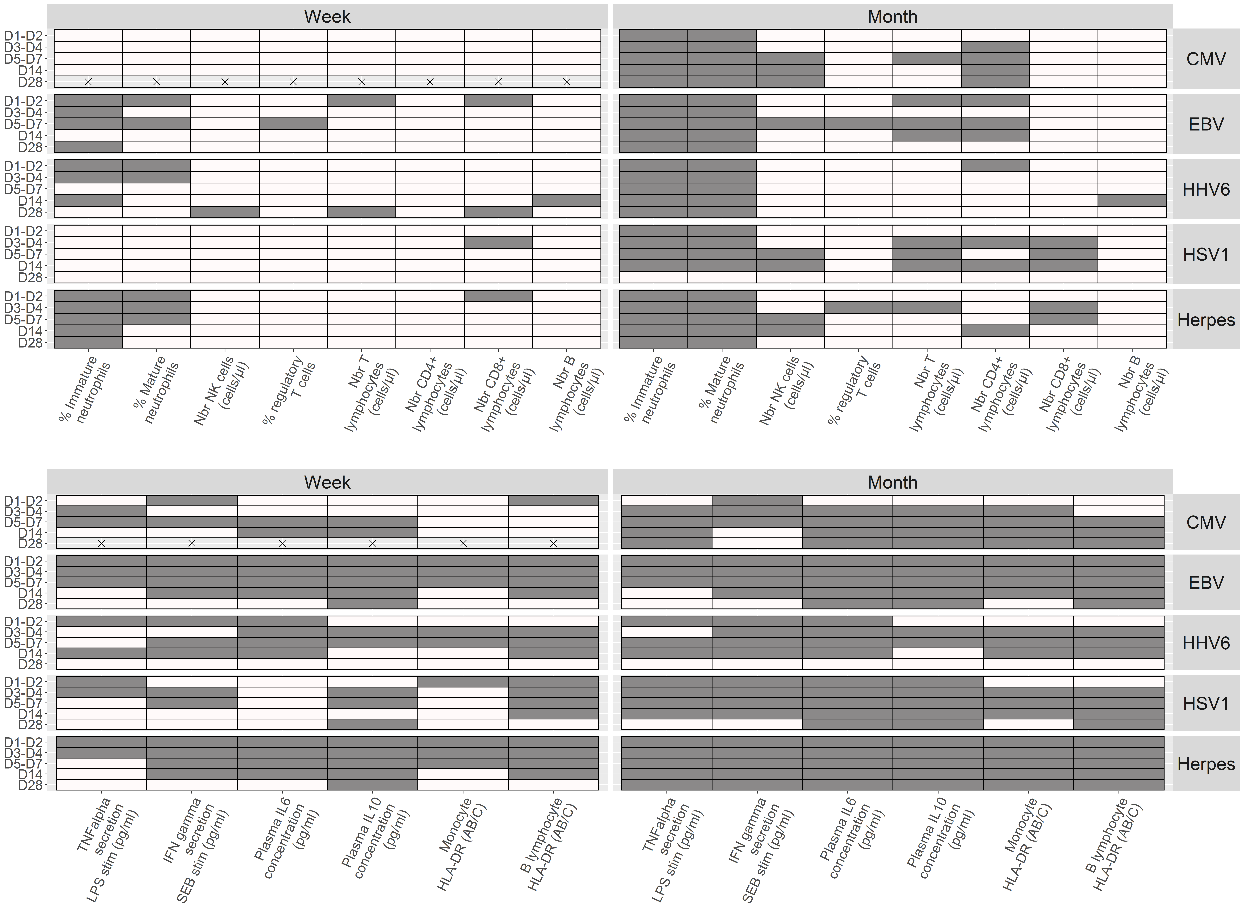** |
| --- | --- | --- |
| **B** | Functional markers |  |
| **C** | mRNA EvaGreen markers | **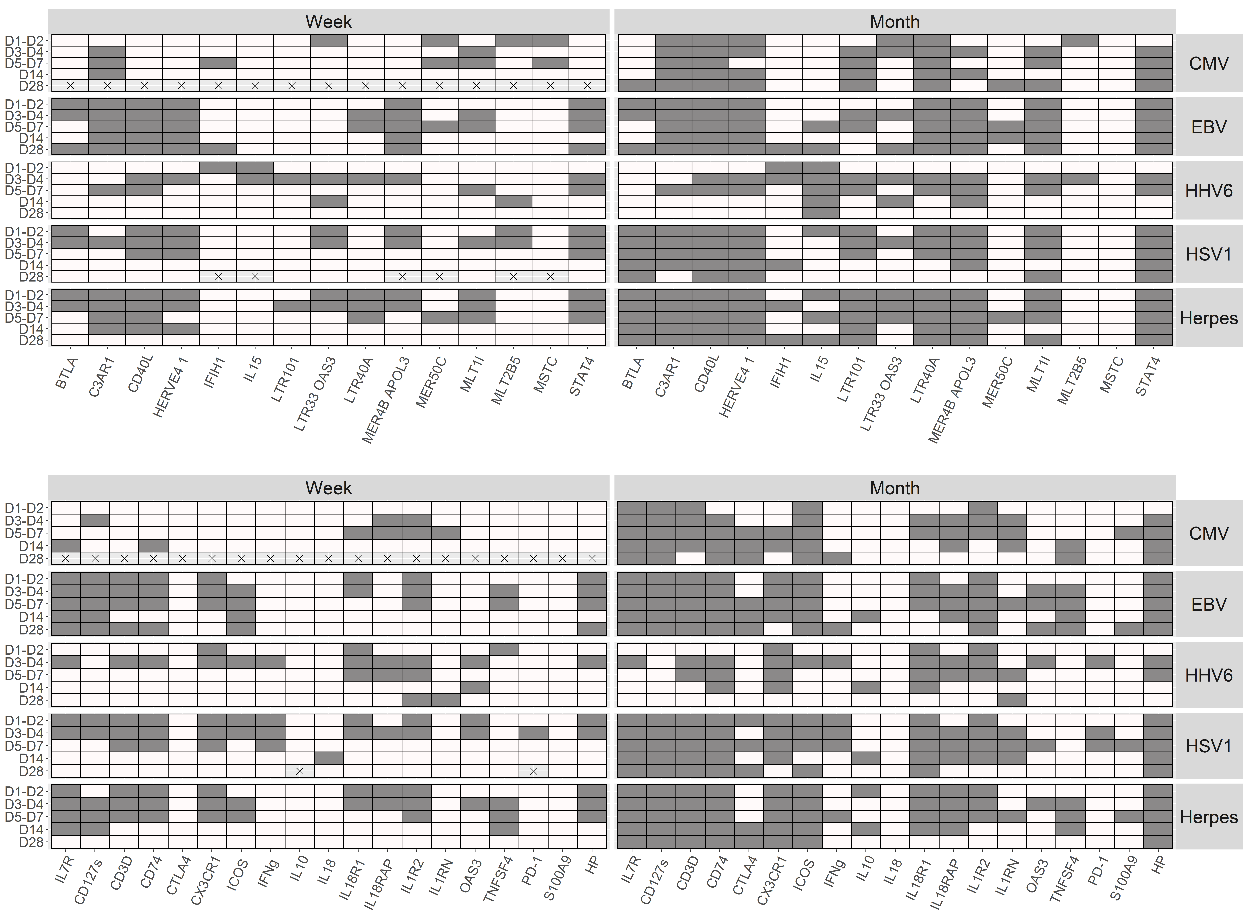** |
| **D** | mRNA TaqMan markers |  |

**Supplementary Figure 2. Association of CMV, EBV, HHV6, HSV1 viremia with immunological and molecular markers.** Wilcoxon tests among global pathological condition were performed to compare markers distribution at different time point classes (D1-D2, D3-D4, D5-D7, D14, D28) and viremia (CMV, EBV, HHV6, HSV1 and all Herpes) detection status during the first week (D1 to D7) and the first month (D1 to D28). Significant associations between viremia and (**A**) cellular marker, (**B**) functional markers, (**C**) mRNA transcripts detected by RT-PCR_EVaGreen and (**D**) mRNA markers detected by Taqman RT-PCR were indicated as grey boxes. When comparing viremia and markers, the occurrence of less than 9 detection events was considered as a lacking data and indicated as X in white boxes.

**
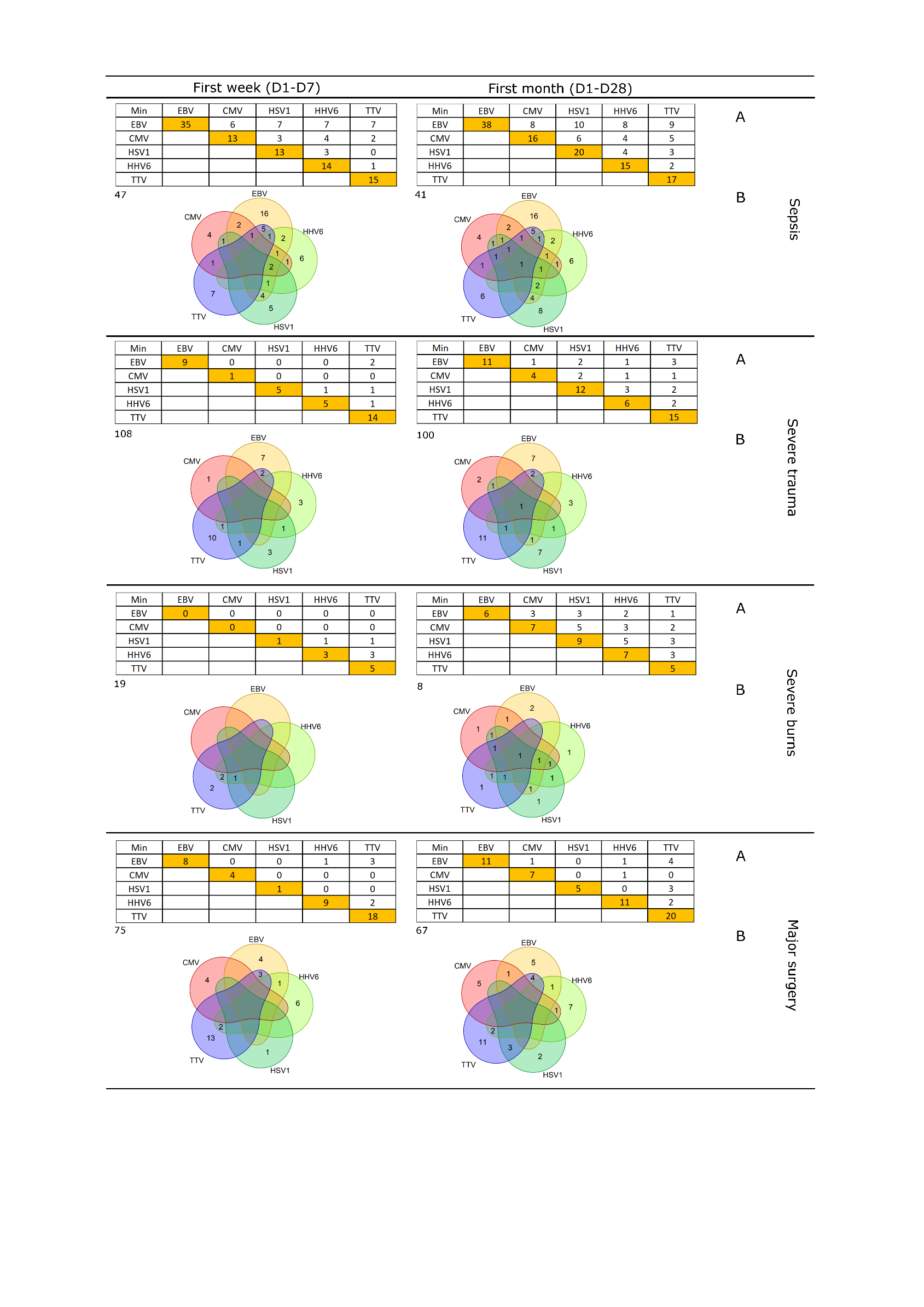
Supplementary Figure 3. DNAemia during the first week and first month following admission, according to pathologies**. **A** Number of patients presenting single- or multiple-positive herpes and/or TTV viral DNAemia during the first 7 days or the first 28 days following admission in the ICU. Occurrence of each type of herpes virus (HV) or TTV is depicted in the orange boxes. Co-occurrence of HV with other HV or TTV is reported in white boxes. **B** Venn diagram illustrating single versus multiple viral reactivations; The number of patients presented with no viral event during the first week and the month is indicated at the top left corner.


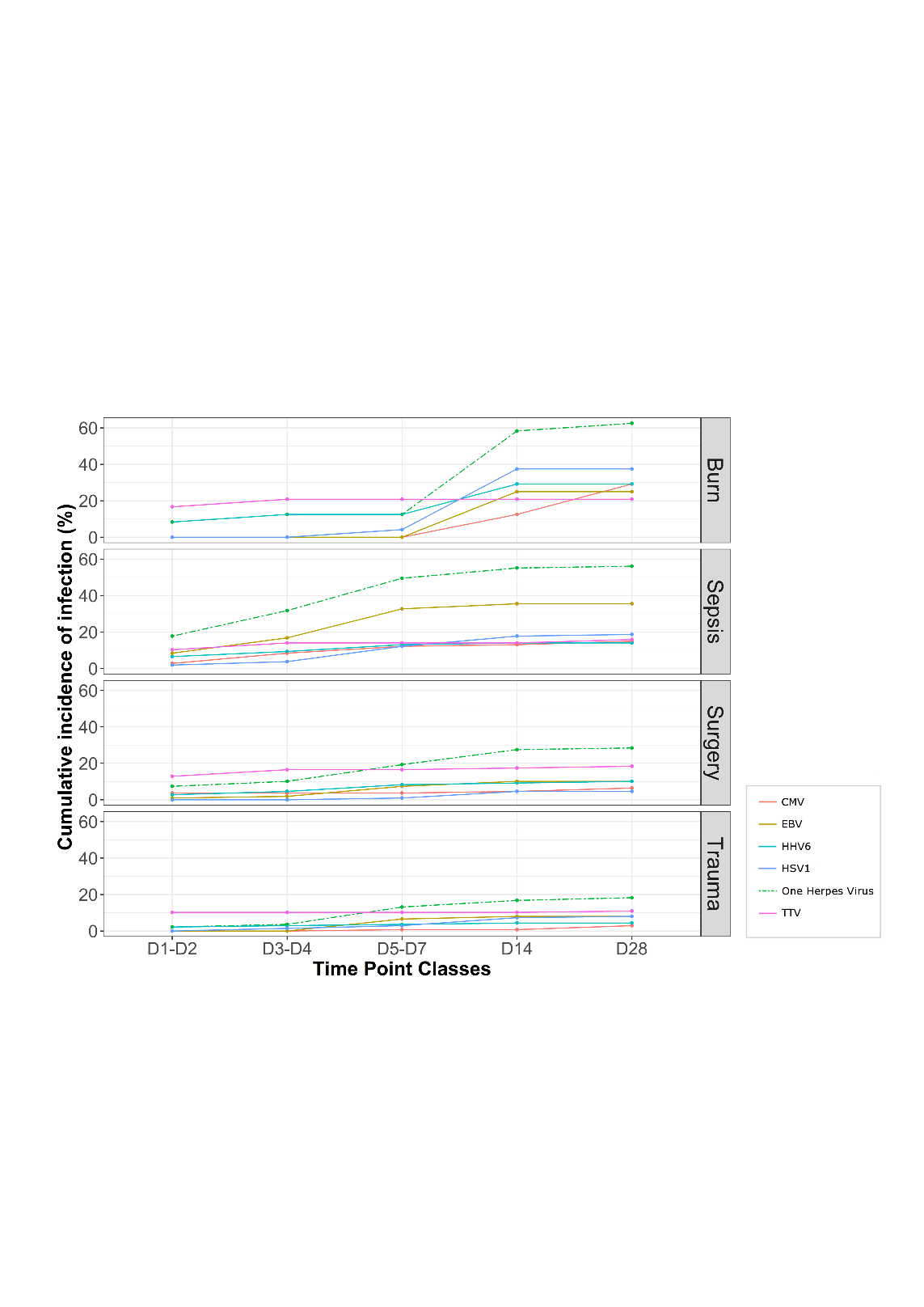


**Supplementary Figure 3 (continued). DNAemia during the first week and first month following admission, according to pathologies**.. **C** Cumulative incidence of individual or collective HV and TTV DNAemia at days 1-2, 3-4, 5-7, 14 and 28, according to pathologies, following admission in the ICU.

**Supplementary Table 3. Comparative plasma DNAemia during the first week and the first month following admission in sepsis patients without IAI versus a group consisting of trauma and surgery and burns patients after IAI**.

| Virus / detection event |  | Sepsis without IAI | |  | Other (Trauma and Burns and Surgery) after IAI | |  | P value Fisher test^C^ |
| --- | --- | --- | --- | --- | --- | --- | --- | --- |
|  |  | DNAemia^a^ | No DNAemia^b^ |  | DNAemia^a^ | No DNAemia^b^ |  |  |
| EBV / first week |  | 24 | 48 |  | 7 | 71 |  | <0.001* |
| EBV / month |  | 26 | 46 |  | 17 | 61 |  | 0.07 |
| CMV / first week |  | 9 | 63 |  | 2 | 76 |  | 0.027* |
| CMV / month |  | 10 | 62 |  | 15 | 63 |  | 0.511 |
| HSV1 / first week |  | 10 | 62 |  | 4 | 74 |  | 0.091 |
| HSV1 / month |  | 12 | 60 |  | 15 | 63 |  | 0.832 |
| HHV6/ first week |  | 5 | 67 |  | 7 | 71 |  | 0.767 |
| HHV6 / month |  | 6 | 66 |  | 12 | 66 |  | 0.216 |

^a^ number of viremic patients in sepsis condition without IAI (only primary infection) versus other conditions (surgery and trauma and burns) post IAI

^b^ number of non viremic patients (negative DNAemia) in sepsis condition without IAI (only primary infection) versus other conditions (surgery and trauma and burns) post IAI

^c^ P value obtained by comparing the number of patients with positive and negative DNAemia in sepsis group versus a group (other) consisting of trauma, burns and surgery patients; p values significant (*) when <0.05

**Supplementary Table 4**: **Association between sepsis primary infection type and herpes virus DNAemia and TTV viremia, according to viral titre, unique viremia or with herpes virus coinfection**.

.

| Virus / detection event | Condition | | Community acquired ^a^ | | Hospital acquired ^b^ | | Number of patients | | pval Fisher test | |
| --- | --- | --- | --- | --- | --- | --- | --- | --- | --- | --- |
| EBV / first week | sepsis without IAI (month) ^c^ | | 30% | | **33%** | | 87 | | 0.8 | |
|  | sepsis (all) ^d^ | | 30% | | **39%** | | 107 | | 0.5 | |
|  |  | |  | |  | |  | |  | |
| EBV / month | sepsis without IAI (month) | | 30% | | **41%** | | 87 | | 0.34 | |
|  | sepsis (all) | | 32% | | **45%** | | 107 | | 0.19 | |
| CMV / first week | sepsis without IAI (month) | | 7% | | **30%** | | 87 | | 0.01 | |
|  | sepsis (all) | | 7% | | **26%** | | 107 | | 0.01 | |
|  |  | |  | |  | |  | |  | |
| CMV / first month | sepsis without IAI (month) | | 8% | | **30%** | | 87 | | 0.02 | |
|  | sepsis (all) | | 11% | | **26%** | | 107 | | 0.07 | |
| HSV1 / first week | sepsis without IAI (month) | | 10% | | **22%** | | 87 | | 0.18 | |
|  | sepsis (all) | | 9% | | **19%** | | 107 | | 0.19 | |
|  |  | |  | |  | |  | |  | |
| HSV / month | sepsis without IAI (month) | | 15% | | **22%** | | 87 | | 0.54 | |
|  | sepsis (all) | | 17% | | **23%** | | 107 | | 0.59 | |
| HHV6 / first week | sepsis without IAI (month) | | 8% | | **19%** | | 87 | | 0.27 | |
|  | sepsis (all) | | 12% | | **16%** | | 107 | | 0.54 | |
|  |  | |  | |  | |  | |  | |
| HHV6 / month | sepsis without IAI (month) | | 10% | | **19%** | | 87 | | 0.S2 | |
|  | sepsis (all) | | 13% | | **16%** | | 107 | | 0.76 | |
| TTVh^e^ / first week | sepsis without IAI (month) | **20%** | | 7% | | 87 | | 0.21 | |  |
|  | sepsis (all) | **17%** | | 6% | | 107 | | 0.22 | |  |
|  |  |  | |  | |  | |  | |  |
| TTVh^e^ / month | sepsis without IAI (month) | **22%** | | 11% | | 87 | | 0.37 | |  |
|  | sepsis (all) | **18%** | | 10% | | 107 | | 0.38 | |  |
| TTVh^e^ only^f^ / first week | sepsis without IAI (month) | **8%** | | 4% | | 87 | | 0.66 | |  |
|  | sepsis (all) | **8%** | | 3% | | 107 | | 0.67 | |  |
|  |  |  | |  | |  | |  | |  |
| TTVh^e^ only^f^ / month | sepsis without IAI (month) | **8%** | | 4% | | 87 | | 0.66 | |  |
|  | sepsis (all) | **7%** | | 3% | | 107 | | 0.67 | |  |
| TTVh^e^ & HpV^g^ / first week | sepsis without IAI (month) | **12%** | | 4% | | 87 | | 0.43 | |  |
|  | sepsis (all) | **9%** | | 3% | | 107 | | 0.43 | |  |
|  |  |  | |  | |  | |  | |  |
| TTVh^e^ & HpV^g^ / first month | sepsis without IAI (month) | **13%** | | 7% | | 87 | | 0.72 | |  |
|  | sepsis (all) | **12%** | | 6% | | 107 | | 0.50 | |  |

^a^ % of patients with a detected viremia according to community acquired primary infection

^b^ % of patients with a detected viremia according to hospital acquired primary infection

^c^ patients without any detectable IAI during the month, i.e. presenting only the primary infection

^d^ all sepsis patients regardless of the occurrence of IAI post primary infection

^e^ TTV DNAemia above 10.000 copies/ml

^f^ exclusively TTV virus

^g^ at least one herpes virus

| **A** |  |  |
| --- | --- | --- |
| IL10 ng/ml | 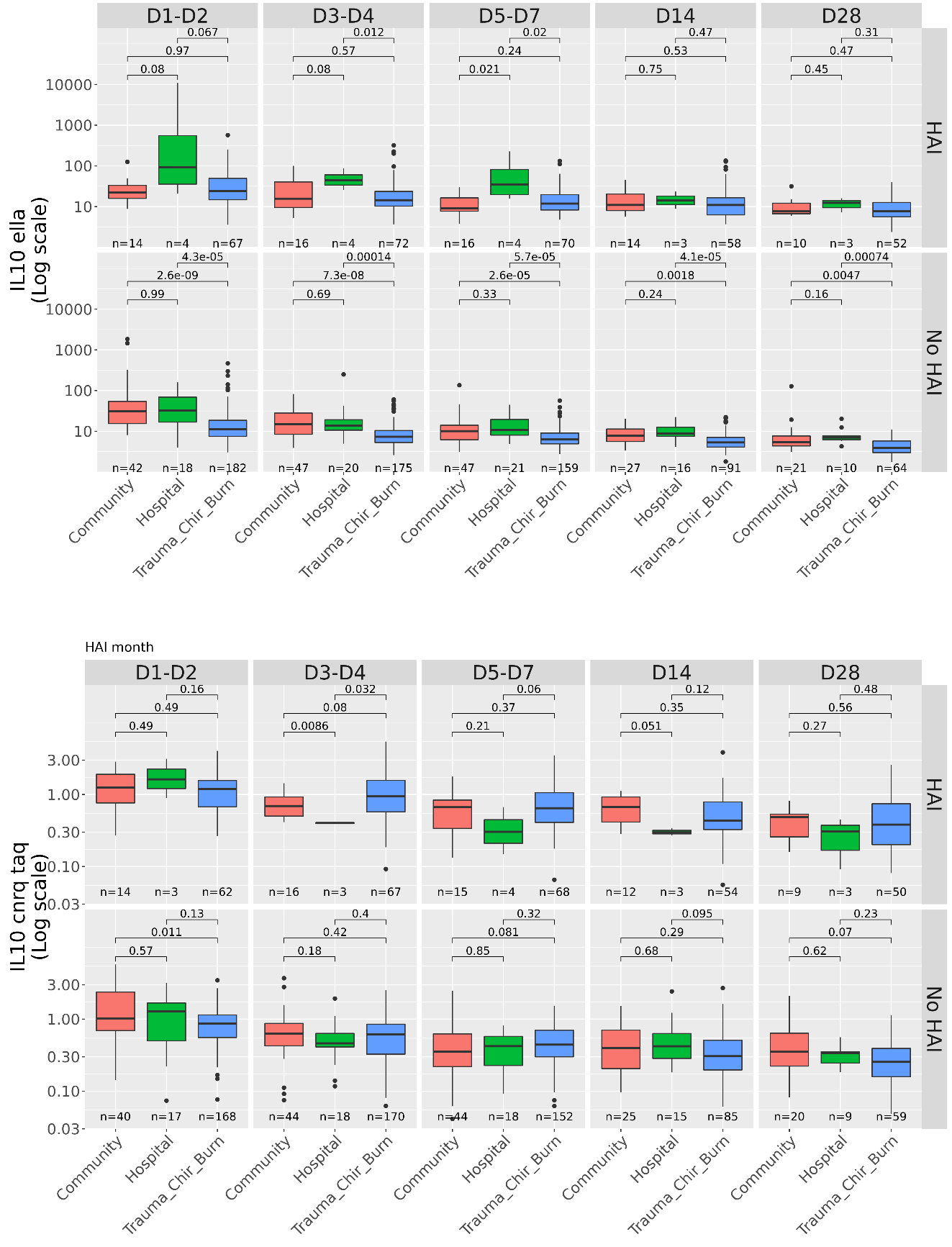 | IAI |
|  |  | No IAI |
|  |  |  |
| **B** |  |  |
| IL10 CNRQ | 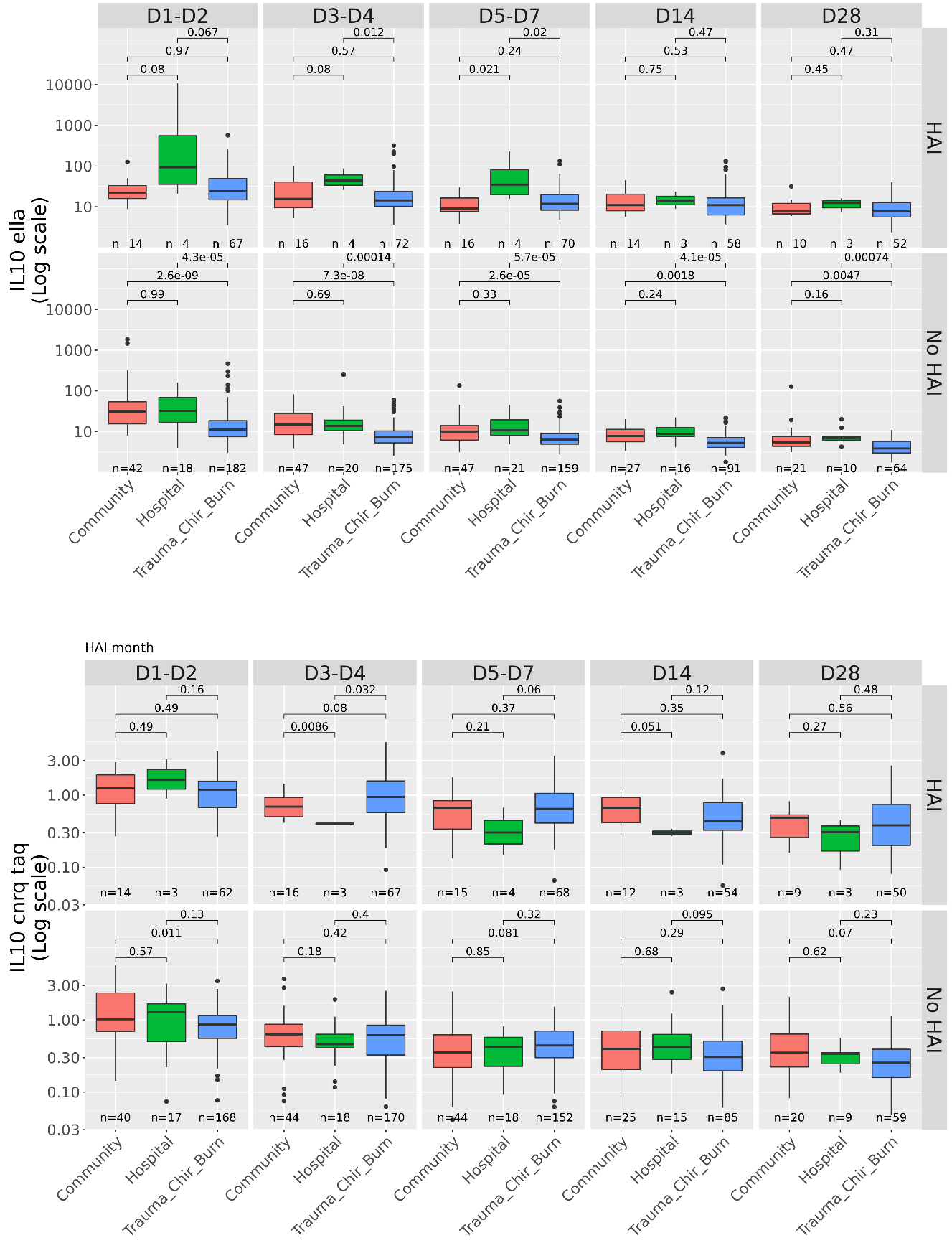 | IAI |
|  |  | No IAI |
|  |  |  |

**Supplementary Figure 4. Comparative IL10 level during the first month following admission in sepsis patients presenting either community (Community) acquired primary infection or hospital (Hospital) acquired primary infection and a group consisting of surgery an trauma and burn patients (Trauma_Chir_Burn).** Patients are further stratified according to the occurrence of IAI event during the month (IAI, no IAI). The level of IL10 as regard to patient groups and time (from D1 to D28) is quantified in serum at the protein level (A) and in whole blood at the mRNA level (B).
